# Transposon-derived transcription factors across metazoans

**DOI:** 10.3389/fcell.2023.1113046

**Published:** 2023-03-07

**Authors:** Krishanu Mukherjee, Leonid L. Moroz

**Affiliations:** ^1^ Whitney Laboratory for Marine Biosciences, University of Florida, St. Augustine, FL, United States; ^2^ Departments of Neuroscience and McKnight Brain Institute, University of Florida, Gainesville, FL, United States

**Keywords:** placozoa, ctenophora, porifera, cnidaria, mollusca, convergent domestication, transcription factors, class II DNA transposons

## Abstract

Transposable elements (TE) could serve as sources of new transcription factors (TFs) in plants and some other model species, but such evidence is lacking for most animal lineages. Here, we discovered multiple independent co-options of TEs to generate 788 TFs across Metazoa, including all early-branching animal lineages. Six of ten superfamilies of DNA transposon-derived conserved TF families (ZBED, CENPB, FHY3, HTH-Psq, THAP, and FLYWCH) were identified across nine phyla encompassing the entire metazoan phylogeny. The most extensive convergent domestication of potentially TE-derived TFs occurred in the hydroid polyps, polychaete worms, cephalopods, oysters, and sea slugs. Phylogenetic reconstructions showed species-specific clustering and lineage-specific expansion; none of the identified TE-derived TFs revealed homologs in their closest neighbors. Together, our study established a framework for categorizing TE-derived TFs and informing the origins of novel genes across phyla.

## 1 Introduction

Transposable elements (TEs) or transposons identified by Barbara McClintock during the 1940-the 50s are now recognized as pivotal regulatory elements (Biemont and Vieira, 2006) controlling roughly 25% of the human genes (Jordan et al., 2003). TEs are also major constituents of all eukaryotic genomes, frequently occupying from 20% to more than 70% of genomes. The inherent ability of TEs to self-replicate, move and mutate transformed the initial assessment of TEs as “selfish gene” parasites and “junk DNA” into powerful evolutionary forces (Miller et al., 1999). The process of genomic integration of TE and thus generating or expanding cis-regulatory elements, genes, and other elements such as micro (microRNAs) or non-coding RNAs (ncRNAs) followed by suppression of parasitic self-propagation properties is called molecular domestication or exaptation (Gould and Vrba, 1982; Miller et al., 1999; Volff, 2006).

A domesticated TE-derived gene regulator can benefit the host and be an adaptive advantage (Miller et al., 1999; Biemont and Vieira, 2006; Volff, 2006; Feschotte and Pritham, 2007). The TE-associated domestication events can be sources of novel genes (Miller et al., 1999), ncRNAs, microRNAs, etc., (Borchert et al., 2011; Li et al., 2011; Chuong et al., 2013; Henaff et al., 2014; Zhang et al., 2016). There are multiple examples of such beneficial domestication events, and the scope of this process is expanding with sequenced genomes (Miller et al., 1999; Jordan et al., 2003; Volff, 2006; Feschotte and Pritham, 2007; Koonin et al., 2020; Sundaram and Wysocka, 2020). There are also examples of convergent domestication, reflecting TE’s nature (Casola et al., 2008; Mateo and Gonzalez, 2014). For example, the emergence of the placenta from the TE-derived *Syncytin* gene in mammals and lizards occurred through two independent occurrences of TE domestication; it is portrayed as a classic example of convergent evolution (Miller et al., 1999; Lavialle et al., 2013; Cornelis et al., 2017).

Perhaps, the most critical domestication episodes associated with the rise of biological novelties are the recruitments of TEs in the evolution of transcription factors (TFs). TFs are known to be master regulators of gene expression across Metazoa (Lewis, 1978; Gehring, 1996), including body patterning (Pearson et al., 2005; Peter and Davidson, 2011) and cell fate commitment (Lin et al., 2010; Vervoort and Ledent, 2001). Mechanisms of the origins and lineage-specific TF gene expansion are primarily unknown. A classical hypothesis implies ancestral TF gene duplication, followed by the divergence of the duplicated gene (Ohno et al., 1968). However, this scenario does not apply to the TFs that are solely organism-specific and have no *bona fide* one-to-one orthologs in closest relatives.

The complementary scenario is the origin of TFs and the novel TF-binding sites with the contribution of TEs. DNA-binding properties of TEs, in particular the evidence that TEs contain TF-binding sites, perfectly match structural genome constraints as a potential “pre-adaptation” and sources to form novel cis-regulatory elements and TFs. Thus, incorporating non-coding and new TF genes into existing transcriptional networks (Sundaram and Wysocka, 2020) can also lead to the origins of new functions and transformative biological innovations, as well as the diversification of both genes and forms.

The most notable examples of TE-derived TFs came from plants (Lin et al., 2007; Henaff et al., 2014) and such model animal species as insects, e.g., *Drosophila* (Miller et al., 1999; Casola et al., 2007; Mateo and Gonzalez, 2014) or vertebrates (Hammer et al., 2005; Cayrol et al., 2007; Balakrishnan et al., 2009; Markljung et al., 2009; Hayward et al., 2013; Majumdar et al., 2013). However, the broad comparative scope of these events is less explored, with little knowledge about the majority of animal phyla.

Practically nothing is known about the most diverse bilaterian lineage–Lophotrochozoa. This clade consists of more than a dozen phyla (Kocot et al., 2017), including Mollusca—the second most species-rich phylum and one of the most diverse groups of animals (Ponder and Linderg, 2008). The evidence of TE domestication events outside Bilateria in four other basal metazoan lineages (Ctenophora, Porifera, Placozoa, and Cnidaria) is also lacking.

Here, we generated a catalog of potentially TE-derived TFs across Metazoa and proposed independent co-option of six out of ten superfamilies of TEs to create hundreds of TFs in all early-branching animal lineages.

## 2 Results and discussion

1. Mosaic distribution and parallel evolution of transposon-derived transcription factors across metazoans

Using tblastn searches against target genomes we first identified and curated a complete dataset of transcription factors (TFs) encoded in representatives of four animal phyla with the sequenced genomes, including two bilaterians (*Aplysia californica* and *Octopus bimaculoides*), one ctenophore (*Pleurobrachia bachei*), a sponge (*Amphimedon queenslandica*), and a placozoan (*Trichoplax adhaerens*). As a query, we used the most completed, annotated, and published dataset of 1,600 TFs encoded in the human genome to represent the deuterostomes clade (Lambert et al., 2018) and 755 predicted sequence-specific TFs in *Drosophila*, the model representative of the Ecdysozoa clade, as the initial queries for the tblastn searches (Shokri et al., 2019). Utilizing these complete and initial datasets, we identified that the sea slug *Aplysia* genome encodes 824 transcription factors. Similarly, using all *Aplysia*, *Drosophila,* and human TFs as queries in tblastn searches against their genomes, we identified the complete repertoire of TFs encoded in the *Octopus bimaculoides,* and the other three (*Trichoplax*, *Amphimedon*, *Pleurobrachia*) basal metazoan genomes.

**FIGURE 1:**
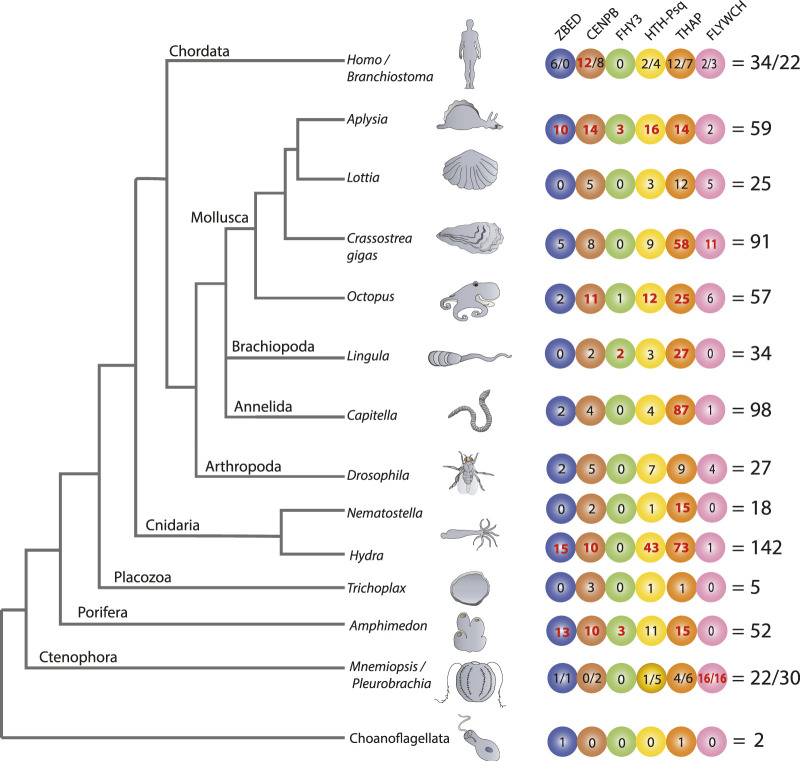
Transposon-derived transcription factors across metazoans. The diagram shows lineage-specific expansion and mosaic distributions of six families of transposon-derived transcription factors (TFs) across metazoans. All TFs depicted in the tree are lineage-specific genes that have no homolog in other classes or phyla. Each colored circle represents one of the six potentially TE-derived TF gene families: ZBED, CNPB, FHY3, HTH-Psq, THAP, and FLYWCH. Figures within circles indicate several independent species-specific events of the domestication of a particular TF family. The total numbers of transposon-derived TFs identified in each reference species are shown on the right. We observed the most extensive expansion of transposon-derived TFs in four bilaterian lineages led to the hydrozoan polyp—*Hydra* (142), the oligochaete—*Capitella* (98), the sea slug—*Aplysia* (59), and the bivalve—*Crassostrea* (91). Of note, a significant expansion of the THAP gene family occurred in *Capitella* (87), *Hydra* (73), and *Crassostrea* (58). Independent species-specific expansions of the FLYWCH gene family occurred in ctenophores *Mnemiopsis* (16) and *Pleurobrachia* (16). The “/” symbol is used to differentiate the numbers identified under both species, such as in *Homo*/*Branchostoma* and *Mnemiopsis*/*Pleurobrachia,* etc., The bold red letter indicates when the values are significantly higher in numbers compared to other species.

**FIGURE 2:**
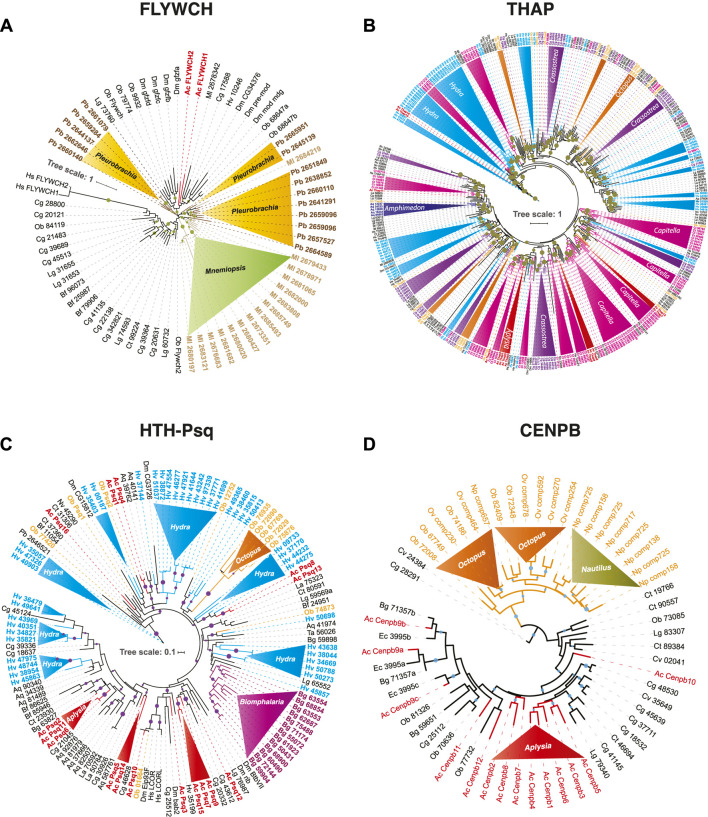
Independent expansion and convergent evolution of transposon-derived transcription factors in Metazoa. The phylogenetic tree represents the independent expansion and evolution of transposon-derived transcription factors protein families across metazoans. Each solid-color triangle represents species-specific expansion that has no homologs in related species. We used the following DNA binding domains–FLYWCH **(A)**, THAP **(B)**, HTH-Psq **(C)**, and CENPB **(D)**—as illustrative examples to build the maximum likelihood (ML) tree. The trees show independent FLYWCH gene expansion in the ctenophores *Mnemiopsis* and *Pleurobrachia* **(A)**. Similarly, independent THAP genes expansion in *Capitella*, *Octopus*, *Crassostrea*, *Hydra* **(B)**, HTH-Psq expansion in *Hydra*, *Biomphalaria*, *Aplysia,* and *Octopus* **(C)**, and Independent convergent domestication of CENPB genes in *Octopus*, *Nautilus,* and *Aplysia* **(A)**. High-resolution images of each of these trees are presented in Supplementary Figures.S1–S4.

**FIGURE 3:**
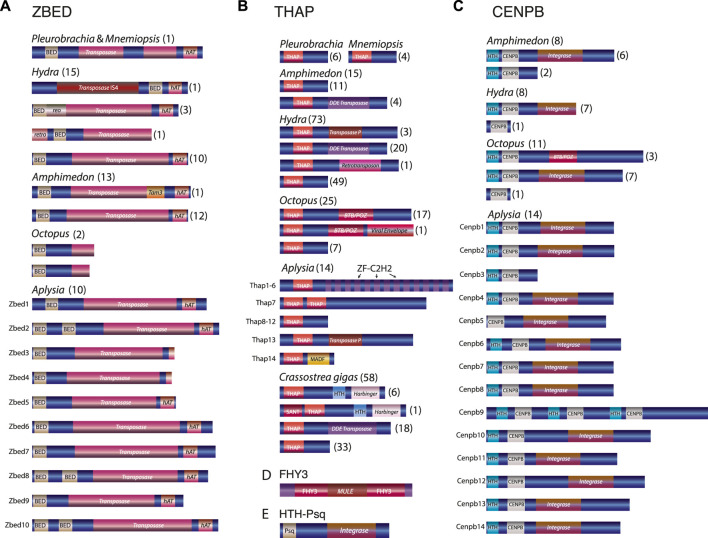
Domain organizations of the transposon-derived transcription factors across metazoans **(A–E)**. Transposon insertions domains are shown in shaded red color and labeled as *integrase*, *transposase*, *Harbinger*, *BTB/POZ,* etc., Note that the same transcription factor protein families have different transposon components. For example, *Octopus* CENPB and THAP proteins have derived mostly from BTB/Poxvirus BTB (Godt et al., 1993)/POZ (Bardwell and Treisman, 1994) transposable elements, whereas, in other species, the same TFs have originated from multiple different transposable elements. Similarly, *Hydra* ZBED genes could have derived from at least three transposon sources such as *retrotransposon*, *reoviruses,* and *transposon IS4,* whereas all *Aplysia* ZBED genes seem to have derived from *Ac* transposon (Supplementary Figures S5, S6). Numbers within parentheses indicate the number of genes identified with a similar domain organization.

Next, we identified TF families in these five animal phyla that have undergone lineage-specific TFs gene expansions, including the ones that have originated through tandem duplications. To our surprise, we found that the full-length TFs that derived from the class II DNA transposable elements (TEs) were primarily associated with species-specific TFs family gene expansion (Figure 1). Within this framework, Cosby et al. (Cosby et al., 2021) not only described the tendency of class II TE for being domesticated as TFs in mammals but also study mechanisms and proposed a model for this process, taking into count the binding sites of transposases. There are ten superfamilies of Class II TEs that are known to use the “*cut-and-paste*” mechanism for transposition from one position in the genome to another (Feschotte and Pritham, 2007; Zattera and Bruschi, 2022). Representatives of each of these subfamilies TE encoded full-length TF proteins were used as a query to screen for potentially TE-derived TFs across nine metazoan phyla (Figure 1; Supplementary Table S1). We determined that six of these TEs superfamilies could be independently recruited into the metazoan TFs: ZBED, CENPB, FHY3, HTH-Psq, THAP, and FLYWCH (Figure 1). Phylogenetic reconstruction suggested independent recruitment due to the absence of a “one-to-one” homolog in the closest species (Figure 2 **)**. The domain organization of newly identified potentially TE-derived metazoan TFs (summarized in Figure 3) also revealed the presence of transposon-like components within the protein-coding open reading frames (ORFs). The occurrence of TEs components within the TFs was further supported by sequence similarity searches against the *de novo* assembled transcriptome (RNA-Seq) dataset (https://neurobase.rc.ufl.edu).

**FIGURE 4:**
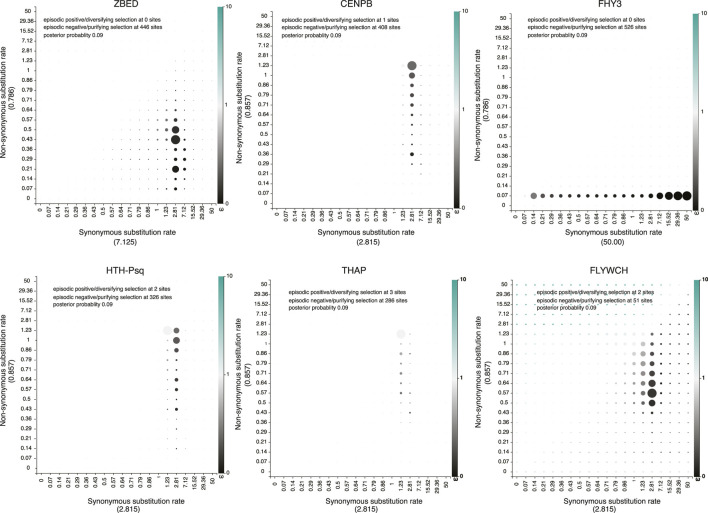
Non-synonymous (dN) versus synonymous substitution (dS) ratio show transposon-derived transcription factors evolving under purifying selection pressure. Non-synonymous *versus* synonymous substitutions were calculated across all potentially TE-derived TF families using the Fast Unbiased Bayesian Approximation (FUBAR) approach (Murrell et al., 2013). Synonymous substitutions (dS) rates calculated under each family are shown in *X*-axis inside the parentheses. Similarly, Non-synonymous substitutions (dN) rates calculated under each family showed in the Y axis inside the parentheses. Gray to intense black color-coding dots signifies negative or purifying (dN/dS < 1) selection, while light green to intense green represents sites under diversifying or positive (dN/dS > 1) selection.

All predicted TE-derived TF families identified in our analysis showed low ( <1; Z-test *p* < 0.05) non-synonymous substitutions *versus* synonymous substitution (Ka/Ks) ratios (Supplementary Excel File S2, S3), indicating negative or purifying selection acting to maintain evolutionarily conserved sets of amino acid sequences. Similarly, the low Ka/Ks ratio of predicted TE-derived TFs suggests stationary domesticated genes (Gao et al., 2020). Furthermore, maintaining low Ka/Ks also suggest that their transposition ability can be maintained (Dazeniere et al., 2022). In addition to the Z test, Fast Unbiased Bayesian Approximation (FUBAR) (Murrell et al., 2013) estimation of the dN/dS ratio also confirmed negative or purifying selection pressure acting on these TFs (Figure 4). The total number of the proposed transposon-derived TFs is 788 (Supplementary Excel File S1). Supplementary Table S3 includes species such as the sea slug, *Elysia chlorotica*, the hemipteran insect *Myzus persicae*, and the rainbow trout *Oncorhynchus mykiss* (Supplementary Excel File S1).

Figure 1 illuminates the mosaic-type distribution in the recruitments of transposon-derived TF subfamilies across major metazoan lineages studied here. In the sister group to all Metazoa—Choanoflagellata—we found only two genes likely encoding transposon-derived TFs from ZBED and THAP superfamilies, respectively.

Ctenophores are often viewed as the earliest branching lineage of animals, sister to the rest of Metazoa (Ryan et al., 2013; Moroz et al., 2014; Whelan et al., 2015; Whelan et al., 2017), although the reconstruction of the basal metazoan phylogeny is still a highly debated topic (Kapli and Telford, 2020; Li et al., 2021; Redmond and McLysaght, 2021), and might not be convincingly resolved. Unlike other studied metazoans, both the ctenophores *Mnemiopsis* and *Pleurobrachia* showed tremendous expansions of the FLYWCH transcription factor gene family (Figure 2A). FLYWCH (Dorn and Krauss, 2003; Ow et al., 2008), which is a distinct DNA-binding zinc finger domain-containing protein family known to have originated from the *Mutator* transposase (Marquez a Pritham, 2010). FLYWCH domains are evolutionary conserved but relatively rarely occur in animals. They were initially identified in *Drosophila* (Dai et al., 2004) and then in *C. elegans*, where it plays regulatory roles during embryogenesis by repressing microRNAs (Ow et al., 2008). The most recent evidence suggests that FLYWCH, in complex with *β*-catenin, repressed specific genes of the Wnt pathways and, therefore, can control cell polarity, migration, and metastasis (Muhammad et al., 2018). Surprisingly, none of the newly identified FLYWCH domain-containing genes have homologs in each other ctenophore species (Figure 2A; Supplementary Figure S1). Unfortunately, there are no functional studies of these genes, and the roles of these TFs in ctenophores will be subjects of future studies.

There are three species with the broadest overall domestication of TEs: the hydroid polyp—*Hydra* (142 TFs), the polychaete annelid—*Capitella* (98 TFs), and the gastropod mollusk, *Aplysia* (59 TFs). In these animals, the identified domestication events are both species-specific and TF-type-specific. In other words, for each animal studied, we noticed an independent expansion of one or more families of potentially TE-derived TFs (Figure 1). The most notable examples of predicted TE exaptation we found in *Hydra* and the ctenophore *Pleurobrachia* (5 out of 6 superfamilies), *Aplysia* (6 out of 6 superfamilies), and the sponge *Amphimedon* (5 out of 6 superfamilies). Surprisingly, the lineage that led to the sponges also revealed multiple examples of independent domestication and expansion of potentially TE-derived TFs compared to other non-bilaterian metazoans (except *Hydra*), which correlate to astonishing diversification within the phylum Porifera in general.

In contrast, the placozoan *Trichoplax*—the simplest known free-living animal (Grell and Ruthmann, 1991; Srivastava et al., 2008; Romanova et al., 2021; 2022), had the smallest number (5) of predicted TE-derived TFs, which might reflect the observed morphological simplicity of these disk-shaped benthic animals with only three layers of cells gliding on algal substrates (Srivastava et al., 2008; Smith et al., 2014; Eitel et al., 2018).

Likewise, the anthozoan *Nematostella* also had a modest representation of potentially TE-derived TFs, mostly related to just one superfamily; there are 15 Thanatos and associated protein (THAP) domain-containing genes. THAP genes were found in *Drosophila,* and they are known to have originated from P element transposes (Roussigne et al., 2003). Our analysis support events of the independent diversification of THAP genes in *Hydra* (73), *Capitella* (87), *Crassostrea* (58) (see details in the next section and Figure 2B; Supplementary Figure S2); and at a lesser degree in a living fossil—the brachiopod, *Lingula* (27) and *Octopus* (25).

In summary, THAP genes represent the largest class of potentially TE-derived TFs identified in this study, including the basally branched chordate amphioxus (*Branchiostoma*) and humans. THAP- TF functions in invertebrates are primarily unknown (Nicholas et al., 2008). On the other hand, THAP TFs in humans were implicated in epigenetic regulation, maintenance of pluripotency, transposition, cancers, and other disorders like hemophilia. For example, THAP0 is a member of the apoptotic cascade induced by IFN-γ (Lin et al., 2002). THAP1, with RRM1, regulates cell proliferation (Cayrol et al., 2007). THAP5 acts as a cell cycle inhibitor (Balakrishnan et al., 2009). THAP9 is an active transposase in humans (Majumdar et al., 2013). The THAP11 homolog in mice is essential for embryogenesis (Dejosez et al., 2008).

Two other groups presently identified TE-derived TFs are also prominent in humans and *Branchiostoma*: ZBED and CENPB (Figure 1; Supplementary Figures S5–S7).

BED zinc fingers or ZBED genes reported having derived from the *hAT* (hobo, Ac, Tam3) superfamily of DNA transposon (Aravind, 2000), and members of this superfamily regulate an extensive array of functions in vertebrates. For example, ZBED6 affects development, cell proliferation, wound healing, and muscle growth (Markljung et al., 2009). ZBEDs are present in mammals, birds, reptiles, and fish; however, they are absent from jawless fishes. Based on these findings, it was proposed that ZBED genes in vertebrates originated due to at least two independent *hAT* DNA transposon domestication events in primitive jawed-vertebrate ancestors (Hayward et al., 2013). Our searches against the *Branchiostoma belcheri* genome uncovered a full-length ZBED gene, which was surprisingly absent from the *Branchiostoma floridae* genome, further suggesting species-specific and mosaic exaptation of TE-encoded genes.

Also, using both the DNA binding BED domain and known full-length ZBED genes, we find that ZBED genes form a monophyletic cluster in three mollusks (*Aplysia*, *Biomphalaria*, *Crassostrea*), the sponge *Amphimedon,* and *Hydra* (Supplementary Figures S5–S6).

Centromere-binding proteins-B (CENPB) transcription factor (Lein et al., 2007) involved in chromosome segregation maintenance and genome stability (Morozov et al., 2017) recurrently domesticated from *pogo*-like transposons (Casola et al., 2008; Mateo and Gonzalez, 2014) across Metazoa (Supplementary Figure S7). CENPB homologs were found in mammals (Sullivan and Glass, 1991) but not in other vertebrates. Nevertheless, we identified CENPB TFs from both *Branchiostoma belcheri* and *B. floridae* genomes, indicating their presence before the divergence of vertebrates. Thus, this finding suggests either loss of CENPBs in most of the extant lineages of vertebrates or their independent domestication in mammalian species, which is a more likely scenario (Casola et al., 2008). There is also a remarkable diversification and independent expansion of the CENPB superfamily in Mollusca (Supplementary Figure S7), which we will discuss in the following section.

The most stunning example of mosaic recruitment of TEs can be illustrated using *Mule* transposons. *Mule* transposon-derived transcription factor far-red elongated hypocotyls 3 (FHY3) group are critical for far-red (near-infrared) light signaling and survival of chloroplast in plants (Lin et al., 2007; Chang et al., 2015). Here for the first time, we identified FHY3 in animals (Figures 1, 3D). Our cross-species comparison across metazoans showed that FHY3 was present in three copies, both in the demosponge *Amphimedon* and the sea slug *Aplysia* genomes. There are two copies in the brachiopod *Lingula* and one in *Octopus* genomes (Figure 1). However, we did not find FHY3 in the sequenced ctenophores (*Pleurobrachia* and *Mnemiopsis*), placozoan (*Trichoplax*), and cnidarian (*Nematostella* and *Hydra*) and human genomes. Thus, FHY3 can be absent or present in a mosaic fashion without a recognized taxonomical specification. Our phylogenetic analysis (Supplementary Excel File S1) showed that FHY3 had been repeatedly domesticated over 550 + million years of animal evolution (see Supplementary Figure 8S), including examples from selected molluscs (e.g., the algae-eating sea slugs *Aplysia californica*, *Elysia chlorotica,* and the oyster—*Crassostrea*), some arthropods (*Myzus persicae* and *Limulus polyphemus*) and chordates (*Branchiostoma*).

In conclusion, we obtained evidence that the majority of TFs are the results of the species-specific convergent domestication events across animal phyla tested here. Figure 2; Supplementary Figures S1–S8 illustrate these cases. Of note, although some of the studied species show a predominant exaptation of just one or two categories of genes, many domesticated events occurred independently, even within the same superfamily of potentially TE-derived TFs (Figure 2; Supplementary Figures S1–S8). This situation is summarized below, focusing on the Lophotrochozoan lineage.

2. Transposon-derived TFs showed independent species-specific expansion and evolution in Molluscs.

Lophotrochozoa or Spiralia, including the phylum Mollusca, is the most morphologically and biochemically diverse animal clade (Kocot et al., 2017). None of the predicted TE-derived TFs were previously reported in Lophotrochozoa (Table 1). The phylum Mollusca in our analysis is represented by seven species (*Aplysia*, *Biomphalaria*, *Elysia*, *Lottia*, *Crassostrea, Octopus,* and *Nautilus*), with *Aplysia* showing the most remarkable expansion of potentially TE-derived TFs (Figure 1). First, we systematically scanned the complete set of the TFs encoded in the *Aplysia californica* genome a prominent neuroscience model (Kandel, 2001; Moroz et al., 2006; Moroz, 2011), resulting in the identification of 824 transcription factors.

**TABLE 1:** The total number of potentially TE-derived TFs identified in this study. (See Figure 1; Supplementary Table S1 for details).

TE-derived TF families	Total numbers identified	Comments on 1st time identification	Top 3–4 species highlighted*
ZBED	71	1st for Lophotrochozoa	*Aplysia* (10), *Amphimedon* (13), ***Hydra*** (**15**)
CENPB	121	1st for Lophotrochozoa	***Aplysia*** (**14**), *Homo* (12)*, Octopus* (7)
FHY3	23	**1**st **for Metazoa**	***Aplysia*** (**3**), *Amphimedon* (**3**), *Lingula* (2), *Octopus* (1)
HTH-Psq	136	1st for Lophotrochozoa	*Aplysia* (16), ***Hydra*** (**43**)*, Octopus* (12)
THAP	370	1st for Lophotrochozoa	***Capitella*** (**87**), *Hydra* (73)*, Crassostrea* (58)
FLYWCH	67	1st for Ctenophora and	Expansion in Ctenophores
		Lophotrochozoa	
	**Total = 788**		

* Topmost 3–4 species that have the highest expansion of TE-derived TFs are shown. The number of TE-derived TFs identified is shown inside the parenthesis. The bold letter is used to highlight the significant increase over other species or the first time detected in the entire metazoan phylogeny.

Then, we identified 59 novel (∼7%) transposon-derived TFs that have no homolog in closely related species such as in *Biomphalaria* the freshwater pulmonated snail (Adema et al., 2017) or the limpet *Lottia* (Simakov et al., 2013)*.* This finding indicates that these TFs did not originate from canonical gene duplication events (Supplementary Excel File S1); they do not follow the canonical subfunctionalization (Stoltzfus, 1999) and neofunctionalization (Force et al., 1999) characteristics. Of these 59 *Aplysia* lineage-specific TFs, 42 were coupled with the transposase (TPase) domain (Figure 3), confirming the hypothesis that these genes, including their DNA-binding domain, may have originated by unique mechanisms involving “*cut-and-paste*” DNA transposons.

In molluscs, we also revealed that the lineage-specific TFs, even those belonging to identical TF families, originated both from similar and different transposon sources: the majority of potentially TE-derived TF domestication events were not detected from related species. Thus, the most likely parsimonious scenario is a broad scope of independent domestication events leading to the convergent evolution of TE-derived TFs within animal lineages studied here. Figure 2; Supplementary Figures S1–S8 illustrates bursts of parallel expansions of transposon-derived TFs subfamilies. Three examples are outlined below.

(1) There are convergent domestications of *pogo*-derived CENPB sequences in *Aplysia*, cephalopods, and other Lophotrochozoan species, such as in *Crassostrea* (Figure 2D). Within the cephalopod lineage, we identified two distinct events of *pogo* domestication—one, in the lineage leading to *Nautilus* and another event occurring in the lineage leading to *Octopus* (Figure 2D).
(2) Helix-turn-helix motif of pipsqueak (HTH-Psq) proteins form a family of transcription factors known to have derived from *Drosophila* pogo transposase (Siegmund and Lehmann, 2002). We find the *Aplysia* genome encodes 16 HTH-Psq subfamily transcription factors while the *Biomphalaria* genome encodes 15. Surprisingly none of these *Biomphalaria* TFs has direct homologs in the *Aplysia* genome and *vice versa* (Figure 2C; Supplementary Figure S3), indicating species-specific expansion event. Similarly, both *Hydra* and *Octopus* showed independent species-specific expansions of transposon-derived HTH-Psq genes. Thus, independent domestication of Psq genes might occur at least five times in *Aplysia*, *Biomphalaria*, *Octopus,* and the *Hydra* and *Amphimedon* genomes (Figure 2C).
(3) Myb-SANT, like in Adf (MADF) domain-containing genes initially identified in *Drosophila* known to have originated from the P instability factor or PIF superfamily of DNA transposon (Lin et al., 2007). We find that MADF genes were expanded in *Amphimedon*, *Drosophila,* and, most of all, *Aplysia* with at least six predicted independent domestication events. Although MADF genes are likely derived from the PIF superfamily of DNA transposon, we have excluded MADF genes from this analysis owing to the growing concern that these genes do not harbor a recognized transposon-derived transposase domain within the protein-coding gene.

Altogether our results suggest a substantial lineage-specific diversification and independent evolution of new genes originating from a modular diversity of *cut-and-paste* DNA transposons, as outlined in the next section.

## 3 Domain analysis revealed the presence of transposons derived components within the protein-coding TFs

All subfamilies of transposon-derived TFs predicted in this analysis have a modular domain architecture (Figure 3). Within each subfamily, most TFs encode recognizable transposon-derived components within exons of these protein-coding genes. For example, transposon-derived ZBED TFs, besides encoding the canonical DNA-binding BED zinc finger motif, also encoded a transposon-derived *transposase* domain and an *hAT* dimerization domain (Figure 3A). Strikingly, we find that ZBED genes across metazoans derived from diverse transposable element components (Supplementary Figures S5, S6). For instance, Homo ZBED5 is known to have derived from Buster DNA transposon (Hayward et al., 2013), which, in our analysis, forms a robust clade with one of the *Octopus* ZBED genes indicating its *Buster* transposon origin (Supplementary Figures S5, S6). In contrast, the second *Octopus* ZBED gene forms a robust cluster with the *Hydra retrotransposon-derived* ZBED gene (Supplementary Figures S5, S6). The two truncated ZBED genes from the *Octopus bimaculoides* genome lack an intact transposase and an *hAT* dimerization domain. In addition, we could not recover the full-length transposase domain and the *hAT* dimerization domain from the *Octopus bimaculoides* genome associated with them. This result indicates that the two *Octopus* ZBED genes may have evolved from two independent transposon components.

Similarly, the *Hydra retrotransposon-derived* ZBED gene encodes an intron that separates the N-terminal reverse transcriptase (RT) domain against the C-terminal BED finger and the transposase domain. This result suggests that the *Hydra* BED and the transposase domains are no longer part of the retrotransposon component. In addition, *Hydra* ZBED genes contained at least three transposon components, such as *retrotransposons*, *reoviruses,* and *transposon* IS4 (Figure 3A; Supplementary Figures S5, S6). Likewise, while *Octopus* THAP genes are mostly derived from BTB (Godt et al., 1993) (Broad- Complex, Tramtrack, Bric a Brac) or POZ (Bardwell and Treisman, 1994) (poxvirus and zinc finger) transposon sources—the *Hydra* THAP genes, however, found to be derived from versatile transposon sources such as *Transposase P* element, *DDE transposase* (*DDE_Tnp_4*) and *retrotransposon*. In contrast, some *Crassostrea gigas* THAP genes contained sequences associated with the *Harbinger*-derived transposon domain (Figure. 3B).

Also, while most of the *Octopus* CENPB TFs were associated with the transposon-derived BTB/POZ domain, none of the genes from another mollusc, *Aplysia*, contained this domain (Figure 3C).

Both CENPB and HTH-Psq genes had a signature of the viral *rve* superfamily of the retroviral integrase domain (Figure 3C, E). Integrase is the retroviral enzyme that catalyzes the integration of virally derived DNA into the host cell’s nuclear DNA, forming a provirus that can be activated to produce viral proteins (Delelis et al., 2008). In the same way, FHY3 genes share remarkable sequence similarities with MURA (Hudson et al., 2003), the transposable element encoded by the *Mutator* element of maize, and the predicted transposase of the maize mobile element *Jittery* (Xu et al., 2004). Both transposons are a member of the Mutator-like elements (*MULE*) (Lisch, 2002) (Figure 3D).

These results, for the first time, indicate that even within the same subfamily of transposon-derived TFs—similar domains have derived from multiple transposon components across the animal kingdom. Together our phylogenetic analysis and the revealed domain organizations suggest that similar domain architecture originated in parallel from numerous transposon resources across phyla.

## 4 Conclusion

By systematic analysis of about seven thousand animal TFs, we have predicted a total of 788 ( >10%) novel DNA transposons-derived TFs across metazoans (Figure 1; Supplementary Excel File S1). Our study was limited to 6 previously known TE-derived TF families used as a query to search for the new domestication events. Although predictably derived from the TE components, we had to exclude the MADF genes from the current analysis owing to the absence of a potential transposase domain.

The *Aplysia* genome encodes 41 MADF genes, and a many of them expressed in developmental stages as well as in specific neuronal populations, suggesting their involvement in the control of cell-specific phenotypes (data not shown) as well as contributing to the very origin of neuronal organizations and diversification events (Erwin, 2009; Mustafin and Khusnutdinova, 2020; Moroz and Romanova, 2021). Homologs of these *Aplysia* MADF genes are missing in the sequenced *Biomphalaria* genome a related gastropod species (Adema et al., 2017; Kocot et al., 2011), which encodes only three of these MADF genes. Thus, careful systematic analysis is needed to identify novel domestication events in the evolution of TE-derived TFs within molluscs.

Overall, predicted TE-derived TFs show mosaic patterns in their distribution with extreme heterogeneity and with a ‘sudden’ appearance in one lineage and, at the same time, found to be ‘missing’ in more closely related species.

Although most studied species predict a predominant exaptation of just one category of genes, many domesticated events might occur independently in evolution, even within the same superfamily of potentially TE-derived TFs (Figure 2).

Our results suggest a substantial lineage-specific diversification and independent origins of new TF genes originated from a broad array and a modular diversity of *cut-and-paste* DNA transposons and related viroid-like elements. Many described TFs preserved the original modular gene organization (Figure 3) and could act as highly dynamic modules shaping the genome-wide reorganization within Metazoa.

## 5 Materials and Methods

### 5.1 Identification of potentially TE-derived TFs

We used representatives of published and confirmed domesticated transposable element-derived TFs protein families from plants and animals as a query (Supplementary Table S2). Both PSI-BLAST, as well as Tblastn searches, were performed using both the command-line version at the NCBI standalone BLAST (version 2.2.18) (Camacho et al., 2009) as well as at the online BLAST web interface (Boratyn et al., 2013; Shi et al., 2018) using default e-value cut off for the online version and 10^−5^ to 10^−10^ cut off for the stand-alone blast to identify all potential homologs. Homologs were detected not solely based on e-value cut-off but other criteria such as coverage statistics, bit score, etc., were considered. Protein sequences recovered from one round of TBLASTN or PSI-BLAST searches were recursively used as queries until no further sequences were detected. Each protein blast hit was manually inspected following multiple sequence alignment (MSA) and validated utilizing several databases including the NCBI conserved domain database (CDD) (Marchler-Bauer et al., 2011), Hmmer (Finn et al., 2011), Pfam (Punta et al., 2011), and SMART (Letunic and Bork, 2018). In the case of the non-availability of the gene model (exome), genome sequences surrounding the coding region were excised, and homology-based gene prediction based on hidden Markov models (HMMs) was performed in FGENESH+ (www.softberry.com) to identify the complete open reading frame. Finally, TE insertions within the TFs were further validated by similarity searches against the *de novo* assembled RNA-Seq (transcriptome) datasets obtained in Moroz lab (https://neurobase.rc.ufl.edu).

### 5.2 Multiple sequence alignment and protein domain identification

Protein functional domains were identified by sequence search of the NCBI conserved domain databases (Marchler-Bauer et al., 2011; Marchler-Bauer et al., 2017). Results were verified *via* sequence searches of the SMART (Letunic and Bork, 2018) and Pfam database (Punta et al., 2011). Also, sequences were aligned in MUSCLE (Edgar, 2004a; Edgar, 2004b) and displayed in clustalX (Larkin et al., 2007) and manually confirmed the domain architecture by examining the sequences based on protein secondary structure analysis and profile alignments. Multiple sequence alignment (MSA) obtained through MUSCLE was used to build the HMMER v3.1b2 (Finn et al., 2011) position-specific scoring matrix (PSM) to search against the reference proteome datasets.

### 5.3 Phylogeny reconstruction

Maximum-likelihood (ML) trees were inferred using PhyML v3.0 (Guindon and Gascuel, 2003; Guindon et al., 2010), with the best-fit evolutionary model identified using the AIC criterion estimated by ProtTest (Abascal et al., 2005). ML phylogenies were performed using the JTT model of rate heterogeneity, estimated proportion of invariable sites, four rate categories, and estimated alpha distribution parameter. Tree topology searches were optimized using the best of both NNI (nearest-neighbor interchanges) and SPR (subtree pruning and regrafting) moves (Hordijk and Gascuel, 2005). Clade support was calculated using the SH-like approximate likelihood ratio test (Anisimova et al., 2011). Unless otherwise mentioned, all phylogenetic trees presented throughout the manuscript show SH-support of 80 or greater. The resulting phylogenetic trees were viewed and edited with iTol version 2.0 (Letunic and Bork, 2007).

### 5.4 Estimation of codon substitution pattern and inference of selective pressure

Protein sequences of potentially TE-derived transcription factors under each family were aligned using MUSCLE (Edgar, 2004a), and the conversion of protein alignments to corresponding nucleotide coding sequences was obtained using PAL2NAL webserver (Suyama et al., 2006). Codon-based tests of neutrality and negative or purifying selection were conducted using MEGA with a Z test by calculating the substitution ratio of the number of non-synonymous substitutions per non-synonymous site (Ka) *versus* synonymous substitution per synonymous sites (Ks) using the Nei-Gojobori method (Nei and Gojobori, 1986). Orthologous sequences with a Ka/Ks value of <1 (Z-test, *p* < 0.05) were defined as having been under purifying selection shown with yellow color (Supplementary Excel files S3, S4).

Of note that the extended methods section is summarized in the Supplementary Method section online.

## Data Availability

The datasets presented in this study can be found in online repositories. The names of the repository/repositories and accession number(s) can be found in the article/Supplementary Material.
