# Supplementary material for: Transposon-derived transcription factors across metazoans
Source: Front Cell Dev Biol. 2023 Mar 7;11:1113046. doi: 10.3389/fcell.2023.1113046 (PMC10027918; doi:10.3389/fcell.2023.1113046)
Supplement: Supplementary file 1 [file DataSheet2.PDF]

Supplementary Methods:

Transposon-derived transcription factors in Metazoa

Krishanu Mukherjee[1*], Leonid L. Moroz[1,2*]

[1]Whitney Laboratory for Marine Biosciences, University of Florida, St. Augustine, FL, 32080, USA; [2]Departments of Neuroscience and McKnight Brain Institute, University of Florida, Gainesville, FL, 32610, USA.

*Corresponding authors

Emails:moroz@whitney.ufl.edu

https://orcid.org/0000-0002-1333-3176

krishanu@ufl.edu

Genome-wide identification and annotation of transcription factors across metazoans

We used experimentally verified DNA binding domains as a probe (query) to retrieve the complete repertoire of the transcription factors encoded in the metazoan genome. For example, using the entire dataset of experimentally verified 1600 transcription factors encoded in the human genome as a query(Lambert et al., 2018), we manually curated, annotated, and identify that the *Aplysia* genome encodes 824 transcription factors (Supplementary Excel File 2S). Similarly, the full dataset of transcription factors from both sea slug *Aplysia* and humans was used to retrieve a complete set of transcription factors from *Octopus bimaculoides* and other metazoans such as sponge *Amphimedon*, *Trichoplax,* and ctenophore *Pleurobrachia,* etc. (data not shown). While annotating the

Aplysia genome for the transcription factors we noticed that the *Aplysia* genome encodes a substantial number of the transposon (TE) derived transcription factors (TFs) (100/824 = ~13%), which prompted us to screen the presence of TE-derived TFs across metazoans.

Identification of transposable element-derived transcription factors

We performed comprehensive searches for the transposable element (TEs) -derived transcription factors (TFs) in representatives of all five metazoan lineages such as Ctenophora, Porifera, Placozoa, Cnidaria, and Bilateria, including its three superclades (Deuterostomia, Escdysozoa, and Lophotrochozoa)(Kocot et al., 2017; Moroz et al., 2014; Whelan, Kocot, Moroz, & Halanych, 2015; Whelan et al., 2017). The supplementary Table 1S summarizes the species used in this study and their reference genomes.

Table 1S: Species used in comparative analyses

Species Name	GenBank Genome Assembly / WGS*	References
Monosiga brevicollis	GCA_000002865.1<br>INSDC: ABFJ00000000.1	(King et al., 2008)
Pleurobrachia bachei	GCA_000695325.1<br>INSDC: AVPN00000000.1	(Moroz et al., 2014)
Mnemiopsis leidyi	GCA_000226015.1<br>INSDC: AGCP00000000.1	(Ryan et al., 2013)
Amphimedon queenslandica	GCA_000090795.1<br>INSDC: ACUQ00000000.1	(Srivastava et al., 2010)
Trichoplax adhaerens	GCA_000150275.1<br>INSDC: ABGP00000000.1	(Srivastava et al., 2008)
Hydra vulgaris	GCA_000219015.1<br>GCA_000004095.1	(Chapman et al., 2010)
Nematostella vectensis	GCA_000209225.1<br>INSDC: ABAV00000000.1	(Putnam et al., 2007)
Drosophila melanogaster	GCA_000001215.4	(Adams et al., 2000)

Capitella teleta	GCA_000328365.1<br>INSDC: AMQN00000000.1	(Simakov et al., 2013)
Lingula anatina	GCA_001039355.2<br>INSDC: LFEI00000000.2	(Luo et al., 2015)
Octopus bimaculoides	GCA_001194135.1<br>INSDC: LGKD00000000.1	(Albertin et al., 2015)
Crassostrea gigas	GCA_902806645.1	(Zhang et al., 2012)
Lottia gigantea	GCA_000327385.1<br>INSDC: AMQO00000000.1	(Simakov et al., 2013)
Aplysia californica	GCA_000002075.2<br>INSDC: AASC00000000.3	
Branchiostoma floridae	GCA_000003815.1<br>INSDC: ABEP00000000.2	(Holland et al., 2008)
Homo sapiens	GCA_000001405.28	(Lander et al., 2001)

*WGS—Whole Genemome Shotgun, GCA—(GenBank) genome assembly, INSDC—Internationational Nucleotide Sequence Database Collaboration.

We used representatives of published and confirmed domesticated transposable element-derived TFs protein families from both plants and animals as a query (Supplementary Table 2S). Both PSI-BLAST, as well as Tblastn searches, were performed using both the command-line version at the NCBI standalone BLAST (version 2.2.18)(Camacho et al., 2009) as well at the on-line BLAST web interface(Boratyn et al., 2013) (Shi et al., 2018)using default e-value cut off for the online version and 10^{-5} to 10^{-10} cut off for the stand-alone blast to identify all potential homologs. Homologs were detected not solely based on e-value cut off but other criteria such as coverage statistics, bit score, etc. were considered. Protein sequences recovered from one round of TBLASTN or PSI-BLAST searches were recursively used as queries until no further sequences were detected. Each protein blast hit was manually inspected following multiple sequence alignment (MSA) and validated utilizing several databases including the NCBI conserved domain database (CDD)(Marchler-Bauer et al., 2011), Hmmer(Finn, Clements, & Eddy, 2011),

Pfam(Punta et al., 2011) and SMART(Letunic & Bork, 2018). In the case of non-availability of the gene model (exome), genome sequences surrounding the coding region were excised, and homology-based gene prediction based on hidden Markov models (HMMs) was performed in FGENESH+ (www.softberry.com) to identify the complete open reading frame. Finally, TE insertions within the TFs were further validated by similarity searches against the *de-novo* assembled RNA-Seq (transcriptome) datasets obtained in Moroz lab (https://neurobase.rc.ufl.edu).

Table 2S: Experimentally verified and published transposon-derived transcription factor proteins were used as a query to search against the metazoan genomes.

TF	NCBI Accession	Notes	FastA sequence
ZNF-BED (ZBED)(Hayward, Ghazal, Andersson, Andersson, & Jern, 2013)	NP_004720	All human ZBED genes harboring BED finger, *haT* DNA transposon catalytic and dimerization domain were used as a query to retrieve metazoan ZBED genes. Only one (ZBED1) is shown here	>Hs_ZBED1<br>MENKSLESSQTDLKLVAHPRAKSKVWKYFGFDTNAEGCILQWKKIYCRICMAQIAYSGNTSNLSYHLEKN<br>HPEEFCEFVKSNTEQMREAFATAFSKLKPESSQQPGQDALAVKAGHGYDSKKQQELTAAVLGLICEGLYP<br>ASIVDEPTFKVLLKTADPRYELPSRKYISTKAIPEKYGAVREVILKELAEATWCGISTDMWRSENQNRAY<br>VTLAAHFLGLGAPNCLSMGSRCLKTFEVPEENTAETITRVLYEVFIEWGISAKVFGATTNYGKDIVKACS<br>LLDVAVHMPCLGHTLNAGIQQAFQLPKLGALLSRCRKLVEYFQQSAVAMYMLYEKQKQQNVAHCMLVSNR<br>VSWWGSTLAMLQRLKEQQFVIAGVLVEDSNNHHLMLEASEWATIEGLVELLQPFKQVAEMLSASRYPTIS<br>MVKPLLHMLLNTTLNIKETDSKELSMAKEVIAKELSKTYQETPEIDMFLNVATFLDPRYKRLPFLSAFER<br>QQVENRVVEEAKGLLDKVKDGGYRPAEDKIFPVPEEPPVKKLMRTSTPPPASVINNMLAEIFCQTGGVED<br>QEEWHAQVVEELSNFKSQKVLGLNEDPLKWWSDRLALFPLLPKVLQKYWCVTATRVAPERLFGSAANVVS<br>AKRNRLAPAHVDEQVFLYENARSGAEAEPEDQDEGEWGLDQEQVFSLGDGVSGGFFGIRDSSFL<br><br>**Domain organization:** ZF-BED DNA binding domain; Transposase Rnase H; Transposase *haT* dimerization domain
CENPB(Casola, Hucks, & Feschotte, 2008; Mateo & Gonzalez, 2014)	NP_001801	All 12 human CENPB genes harboring DDE transposase domain were used as a query to retrieve metazoan CENPB genes	>Hs_CENPB<br>MGPKRRQLTFREKSRIIQEVEENPDLRKGEIARRFNIPPSTLSTILKNKRAILASERKYGVASTCRKTNK<br>LSPYDKLEGLLIAWFQQIRAAGLPVKGIILKEKALRIAEELGMDDFTASNGWLDRFRRRHGVVSCSGVAR<br>ARARNAAPRTPAAPASPAAVPSEGSGGSTTGWRAREEQPPSVAEGYASQDVFSATETSLWYDFLPDQAAG<br>LCGGDGRPRQATQRLSVLLCANADGSEKLPPLVAGKSAKPRAGQAGLPCDYTANSKGGVTTQALAKYLKA<br>LDTRMAAESRRVLLLAGRLAAQSLDTSGLRHVQLAFFPPGTVHPLERGVVQQVKGHYRQAMLLKAMAALE<br>GQDPSGLQLGLTEALHFVAAAWQAVEPSDIAACFREAGFGGGPNATITTSLKSEGEEEEEEEEEEEEEEEG<br>EGEEEEEEGEEEEEEGGEGEELGEEEEVEEEGDVDSDEEEEEDEESSSEGLEAEDWAQGVVEAGGSFGAY<br>GAQEEAQCPTLHFLEGGEDSDSDSEEEDDEEEDDEDEDDDDDEEDGDEVPVPSFGEAMAYFAMVKRYLTS<br>FPIDDRVQSHILHLEHDLVHVTRKNHARQAGVRGLGHQS<br><br>**Domain organization:** CENPB N-terminal DNA binding domain; CENPB DNA binding domain; Transposase DDE_1 supernuclease; CENPB dimerization domain
FHY3(Hudson, Lisch, & Quail, 2003)	XP_005107058	*Arabidopsis* FHY3A and FHY3B *MULE* transposon derived TF were used as a query to retrieve metazoan FHY3 genes	>At_FHY3B<br>MTDASQFPAADDTVKMEDDTSCLAITVGQTFNSFSEVKELLNKREEKLFERLVMGRGSEQVSYANQRLKG<br>LSKYADCLVYRKVTFRCKHEGEFKPRGTSKLSTSLKKGCPVKVSLSAERKHNRLIVNSVCSDHNHELFPQ<br>SVGVYPEDRAFTDIEAADIKRLLDFGVAVPKIVNYVRTTYGKNVKVKDIHNFRQKAKLVASEGLSEEEQV<br>LQVLQEMVDNEEGRFSITTDSETCCLPIFVQTSRMRKVLQTFPDCLFIDCTYCKNKFAYPIVVFSVVDGD<br>NCGQCVGYGVVRDEQMSTLSSLFGEFVRMNEDVSVKTVVVDKDASEIGALKETMGECDLVLCRFHVCKSL<br>DEAVDKYCEKRVREKMHVICRDLVMSESETDFESGLEKIPLGAFRTYLEKNWLPVRHMWAHHQTKRLVMF<br>GNMTNSFVERHNRTLKTLANSKMSLSEFFRSLLAYHKTEEKKLLHKVIDMHLRAKVFRPNFDAAGPILSQ<br>AYEVLTPYACDELAHQLNKLHSTKCRYCVESKSIVTLGSADEIRDEVPVDGWTCKCPYFIEKTMPCWHLL<br>ALCQHQNVCPVELFPARFRKQSLLDCCVSDVGEVSSGSNNQVQVVLKKVQSELEKRDVMKEACRELVAVG<br>VNCGQEEFERRISVLHELTCGWRNGENGS<br><br>**Domain organization:** Transposase DDE_Tnp_ISL3 (*MULE*)
HTH-Psq(Siegmund & Lehmann, 2002)	XP_005094672	*Drosophila* pipsqueak domain was used to retrieve HTH-Psq genes from the metazoans	>Ac_Psq1<br>MHAMATTTKKKKRLNYSEESMIAACDCVSKGMMLREAARTHGVPYPTLRARVNGRSSIRQIDRTVLSREQENRLAEWLTE<br>SSRRGFRRTKKQLLSTVQKCLNFNAETTVFKDNMPGEKWYRLFRERHKNKLSQQTPLALGSQRAAVNESKILTWFQKAKK<br>DISQVDLTVLECPERLFNCDESGFQLGGGVRKVLAATTRTDKRVSQVTNDTHKQVTVLVCGNALGELQAPLLIFPGQRFTY<br>NPLEGFEEAHMAKSRNGCIDCEILARWMETAFCPAVAHLQKPVVLFADGNSFHLTLEIHEICRSHGVILYQLPSLNHSSRIVQ<br>PLDLTSFKNLKHAWYEEVMKYQEDNSDTLSKQHFAKVFKSAWDRRGHDGDVLVNGFRASGIFPWNPDRFDRTKLAPPRM<br>FQTVQTVDSTVTAETNGPTVTEPTNEPTIIEYEDETTVTEEPIIEVEQTEIIIDQTETAKDTDVGPSAASHDHNMTVQTAFDRL<br>LHLTARAGSSGLVHYMTCYDLDKTTDEPEQQLFSKLYGRIQTQLVTGLTDTNKDKTAHRPTIFYNLPLPARWGRKKRTVID<br>PPEMVSSDEFNISEENLEKKRRDEEKKKKKRKKIDREEKRLKNIGEKEKRPRV

			**Domain organization:** HTH-Psq DNA binding domain; *Integrase* core domain (DNA copy of the viral genome)
THAP(Quesneville, Nouaud, & Anxolabehere, 2005; Roussigne et al., 2003)	NP_078948	Human THAP9 gene harboring a THAP domain and Transposase_P_element domain was used as query to retrieve metazoan THAP domain protein containing genes	>Hs_THAP9<br>MTRSCSAVGCSTRDTVLSRERGLSFHQFPTDTIQRSKWIRAVNRVDPRSKKIWIPGPGAILCSKHFQESD<br>FESYGIRRKLKKGAVPSVSLYKIPQGVHLKGKARQKILKQPLPDNSQEVATEDHNYSLKTPLTIGAEKLA<br>EVQQMLQVSKKRLISVKNYRMIKKRKGLRLIDALVEEKLLSEETECLLRAQFSDFKWELYNWRETDEYSA<br>EMKQFACTLYLCSSKVYDYVRKILKLPHSSILRTWLSKCQPSPGFNSNIFSFLQRRVENGDQLYQYCSLL<br>IKSMPLKQQLQWDPSSHSLQGFMDFGLGKLDADETPLASETVLLMAVGIFGHWRTPLGYFFVNRASGYLQ<br>AQLLRLTIGKLSDIGITVLAVTSDATAHSVQMAKALGIHIDGDDMKCTFQHPSSSSQQIAYFFDSCHLLR<br>LIRNAFQNFQSIQFINGIAHWQHLVELVALEEQELSNMERIPSTLANLKNHVLKVNSATQLFSESVASAL<br>EYLLSLDLPPFQNCIGTIHFLRLINNLFDIFNSRNCYGKGLKGPLLPETYSKINHVLIEAKTIFVTLSDT<br>SNNQIIKGKQKLGFLGFLLNAESLKWLYQNYVFPKVMPFPYLLTYKFSHDHLELFLKMLRQVLVTSSSPT<br>CMAFQKAYYNLETRYKFQDEVFLSKVSIFDISIARRKDLALWTVQRQYGVSVTKTVFHEEGICQDWSHCS<br>LSEALLDLSDHRRNLICYAGYVANKLSALLTCEDCITALYASDLKASKIGSLLFVKKKNGLHFPSESLCR<br>VINICERVVRTHSRMAIFELVSKQRELYLQQKILCELSGHINLFVDVNKHLFDGEVCAINHFVKLLKDII<br>ICFLNIRAKNVAQNPLKHHSERTDMKTLSRKHWSSVQDYKCSSFANTSSKFRHLLSNDGYPFK<br><br>**Domain organization:** THAP Domain; Transposase *P* element
MADF(Kapitonov & Jurka, 2004)	NP_724481	*Harbinger* transposons encode two distinct proteins: a transposase (Tnp) and a DNA binding protein (MADF). MADF proteins were used as a probe to recover metazoan MADF genes	>Dm_Adf<br>MDKLDANLEQQFDLNLIEAVKLNPVIYDRSHYNYKHFVRKAQTWKQIAETLGVPEQKCTKRWKSLRDKFA<br>REMKLCQESRWRYFKQMQFLVDSIRQYRESLLGKCANGSQSANQVADPSQQQQAQQQTVVDIFAQPFNGS<br>ATTSAQALTHPHEITVTSDAQLATAVGKDQKPYFYEPPLKRERSEEEHSDNMLNTIKIFQNNVSQAVSAE<br>DQSFGMVVTDMLNTLGVRQKAEAKVHIIKYLTDMQLLAQHNKY<br><br>**Domain organization:** MADF DNA binding domain
FLYWCH	XP_014769500	FLYWCH gene harboring both DNA binding domain and the *MULE* derived domain were used as a probe to recover metazoan FLYWCH genes	>Ob_68647<br>MAEFLRSDKGNPLLLFNGYLFVKEKEVGNRRYWKCQNYNKHCKCRAKTAGGEIISVSREHNHTANPAKVE<br>ARRITETVKTEPEGTYNSPQFVISNAASDNSIVTTPVLPLYNHTVIPANVVAPQLTGKVKIELEETCDSS<br>PQQVVVVSTKASDNSFATTPVLPSVSNMKRTIQLVPREQRDKCSKEFPEGPTKAKKAKFVQSEKGKPMLL<br>VDGYLFVKDKELKNKKYWKCQNYKKYCKCRAKTDGDEVISVSGEHNHAGNPVNVEVRRFMEKIKNDSKET<br>RDSPQYVILNAASDISNLTVPALPPLSSIKRTIRRVRQREICGLPVPNHRKDITFPDEYTKTNRGDDFLL<br>FDSGPSDDRMLIFSTRQNLSVLDSCQNVFMDGTFKTVPVIFDQLYTIQGLKNGFCLPLIFGLLPNRKEET<br>YIKFVKTLKTLVPFSNIDSITTDFEPTMIKAVRTEFDSVNLYGSFFHLGHCLYKKVCAFDLKEKYDTDAH<br>FSLSIRMLLALAFVPTEKVYEAFGALVDGAVYPPEALPVVDYFEDTWLGRPCIRNGRRPPIFDLKMWSCF<br>DRMQQDLPETNNAIEVWHRAFLHQVSANRSTLWKFLEELKREQSLNEINIEKLLSGMECETNTKECRISA<br>KQLKKLTESFQEYTNVVDYLCAVAHNVRL<br><br>**Domain organization:** FLYWCH DNA binding domain; Transposase DDE_Tnp_ISL3 (*MULE*)

Species abbreviation used: Hs-*Homo sapiens*, At-*Arabidopsis thaliana*, Ac-*Aplysia californica*, Dm-*Drosophila melanogaster*, Ob-*Octopus bimaculoides*. **Note:** that the transposon derived domlabeled labelled with either pink or red and DNA-binding domains are labeled with cyan or green color. The gray color is used to represent the protein dimerization domain.

Multiple alignment and protein domain identification

Protein functional domains were identified by sequence search of the NCBI conserved domain databases(Marchler-Bauer et al., 2017; Marchler-Bauer et al., 2011). Results were verified via sequence searches of the SMART(Letunic & Bork, 2018) and Pfam database(Punta et al., 2012). Also, sequences were aligned in MUSCLE(Edgar, 2004a, 2004b) and displayed in clustalX(Larkin et al., 2007) and manually confirmed the domain architecture by examining the sequences based on protein secondary structure analysis and profile alignments. Multiple sequence alignment (MSA) obtained through

MUSCLE was used to build the HMMER v3.1b2(Finn et al., 2011) position-specific scoring matrix (PSM) to search against the reference proteome datasets.

Phylogeny

Maximum-likelihood (ML) trees were inferred using PhyML v3.0(Guindon et al., 2010; Guindon & Gascuel, 2003), with the best-fit evolutionary model identified using the AIC criterion estimated by ProtTest(Abascal, Zardoya, & Posada, 2005). ML phylogenies were performed using the JTT model of rate heterogeneity, estimated proportion of invariable sites, four rate categories, and estimated alpha distribution parameter. Tree topology searches were optimized using the best of both NNI (nearest-neighbor interchanges) and SPR (subtree pruning and regrafting) moves(Hordijk & Gascuel, 2005). Clade support was calculated using the SH-like approximate likelihood ratio test(Anisimova, Gil, Dufayard, Dessimoz, & Gascuel, 2011). Unless otherwise mentioned, all phylogenetic trees presented throughout the manuscript showing SH-support of 80 or greater. The resulting phylogenetic trees were viewed and edited with iTol version 2.0(Letunic & Bork, 2007).

Codon substitution pattern and inference of selective pressures

Protein sequences of TE-derived transcription factors under each family were aligned using MUSCLE(Edgar, 2004a) and the conversion of protein alignments to corresponding nucleotide coding sequences were obtained using PAL2NAL webserver(Suyama, Torrents, & Bork, 2006). Codon-based tests of neutrality and negative or purifying selection were conducted using MEGA with a Z test by calculating the substitution ratio of the number of non-synonymous substitution per non-synonymous site (Ka) versus synonymous substitution per synonymous sites (Ks) using the Nei-Gojobori method(Nei & Gojobori, 1986). Orthologous sequences with a Ka/Ks value of <1 (Z-test, P <0.05) were defined as having been under purifying selection shown with yellow color (Supplementary Excel File 3 & 4S).

Alternatively, we used the Bayesian approach of Fast Unbiased Bayesian Approximation (FUBAR)(Murrell et al., 2013) (datamonkey.org) to infer nonsynonymous (dN) and synonymous (dS) substitution rates on a per-site basis on a given codon alignment and corresponding phylogeny (**Fig. 4**).

References

Abascal, F., Zardoya, R., & Posada, D. (2005). ProtTest: selection of best-fit models of protein evolution. *Bioinformatics, 21* 2104-2105. doi:10.1093/bioinformatics/bti263

Adams, M. D., Celniker, S. E., Holt, R. A., Evans, C. A., Gocayne, J. D., Amanatides, P. G., . . . Venter, J. C. (2000). The genome sequence of Drosophila melanogaster. *Science, 287* 2185-2195. doi:10.1126/science.287.5461.2185

Albertin, C. B., Simakov, O., Mitros, T., Wang, Z. Y., Pungor, J. R., Edsinger-Gonzales, E., . . . Rokhsar, D. S. (2015). The octopus genome and the evolution of cephalopod neural and morphological novelties. *Nature, 524* 220-224. doi:10.1038/nature14668

Anisimova, M., Gil, M., Dufayard, J. F., Dessimoz, C., & Gascuel, O. (2011). Survey of branch support methods demonstrates accuracy, power, and robustness of fast likelihood-based approximation schemes. *Syst Biol, 60* 685-699. doi:10.1093/sysbio/syr041

Boratyn, G. M., Camacho, C., Cooper, P. S., Coulouris, G., Fong, A., Ma, N., . . . Zaretskaya, I. (2013). BLAST: a more efficient report with usability improvements. *Nucleic Acids Res, 41* W29-33. doi:10.1093/nar/gkt282

Camacho, C., Coulouris, G., Avagyan, V., Ma, N., Papadopoulos, J., Bealer, K., & Madden, T. L. (2009). BLAST+: architecture and applications. *BMC Bioinformatics, 10* 421. doi:10.1186/1471-2105-10-421

Casola, C., Hucks, D., & Feschotte, C. (2008). Convergent domestication of pogo-like transposases into centromere-binding proteins in fission yeast and mammals. *Mol Biol Evol, 25* 29-41. doi:10.1093/molbev/msm221

Chapman, J. A., Kirkness, E. F., Simakov, O., Hampson, S. E., Mitros, T., Weinmaier, T., . . . Steele, R. E. (2010). The dynamic genome of Hydra. *Nature, 464* 592-596. doi:10.1038/nature08830

Edgar, R. C. (2004a). MUSCLE: a multiple sequence alignment method with reduced time and space complexity. *BMC Bioinformatics, 5* 113. Retrieved from http://www.ncbi.nlm.nih.gov/entrez/query.fcgi?cmd=Retrieve&db=PubMed&dopt=Citation&list_uids=15318951

Edgar, R. C. (2004b). MUSCLE: multiple sequence alignment with high accuracy and high throughput. *Nucleic Acids Res, 32* 1792-1797. Retrieved from http://www.ncbi.nlm.nih.gov/entrez/query.fcgi?cmd=Retrieve&db=PubMed&dopt=Citation&list_uids=15034147

Finn, R. D., Clements, J., & Eddy, S. R. (2011). HMMER web server: interactive sequence similarity searching. *Nucleic Acids Res, 39* W29-37. doi:10.1093/nar/gkr367

Guindon, S., Dufayard, J. F., Lefort, V., Anisimova, M., Hordijk, W., & Gascuel, O. (2010). New algorithms and methods to estimate maximum-likelihood phylogenies: assessing the performance of PhyML 3.0. *Syst Biol, 59* 307-321. doi:syq010 [pii]
10.1093/sysbio/syq010

Guindon, S., & Gascuel, O. (2003). A simple, fast, and accurate algorithm to estimate large phylogenies by maximum likelihood. *Syst Biol, 52* 696-704. Retrieved from http://www.ncbi.nlm.nih.gov/entrez/query.fcgi?cmd=Retrieve&db=PubMed&dopt=Citation&list_uids=14530136

Hayward, A., Ghazal, A., Andersson, G., Andersson, L., & Jern, P. (2013). ZBED evolution: repeated utilization of DNA transposons as regulators of diverse host functions. *PLoS One, 8* e59940. doi:10.1371/journal.pone.0059940

Holland, L. Z., Albalat, R., Azumi, K., Benito-Gutierrez, E., Blow, M. J., Bronner-Fraser, M., . . . Holland, P. W. (2008). The amphioxus genome illuminates vertebrate origins and cephalochordate biology. *Genome Res, 18* 1100-1111. doi:gr.073676.107 [pii]
10.1101/gr.073676.107

Hordijk, W., & Gascuel, O. (2005). Improving the efficiency of SPR moves in phylogenetic tree search methods based on maximum likelihood. *Bioinformatics, 21* 4338-4347. doi:10.1093/bioinformatics/bti713

Hudson, M. E., Lisch, D. R., & Quail, P. H. (2003). The FHY3 and FAR1 genes encode transposase-related proteins involved in regulation of gene expression by the phytochrome A-signaling pathway. *Plant J, 34* 453-471. doi:10.1046/j.1365-313x.2003.01741.x

Kapitonov, V. V., & Jurka, J. (2004). Harbinger transposons and an ancient HARBI1 gene derived from a transposase. *DNA Cell Biol, 23* 311-324. doi:10.1089/104454904323090949

King, N., Westbrook, M. J., Young, S. L., Kuo, A., Abedin, M., Chapman, J., . . . Rokhsar, D. (2008). The genome of the choanoflagellate Monosiga brevicollis and the origin of metazoans. *Nature, 451* 783-788. doi:10.1038/nature06617

Kocot, K. M., Struck, T. H., Merkel, J., Waits, D. S., Todt, C., Brannock, P. M., . . . Halanych, K. M. (2017). Phylogenomics of Lophotrochozoa with Consideration of Systematic Error. *Syst Biol, 66* 256-282. doi:10.1093/sysbio/syw079

Lambert, S. A., Jolma, A., Campitelli, L. F., Das, P. K., Yin, Y., Albu, M., . . . Weirauch, M. T. (2018). The Human Transcription Factors. *Cell, 175* 598-599. doi:10.1016/j.cell.2018.09.045

Lander, E. S., Linton, L. M., Birren, B., Nusbaum, C., Zody, M. C., Baldwin, J., . . . International Human Genome Sequencing, C. (2001). Initial sequencing and analysis of the human genome. *Nature, 409* 860-921. doi:10.1038/35057062

Larkin, M. A., Blackshields, G., Brown, N. P., Chenna, R., McGettigan, P. A., McWilliam, H., . . . Higgins, D. G. (2007). Clustal W and Clustal X version 2.0. *Bioinformatics, 23* 2947-2948. doi:btm404 [pii]
10.1093/bioinformatics/btm404
Letunic, I., & Bork, P. (2007). Interactive Tree Of Life (iTOL): an online tool for phylogenetic tree display and annotation. *Bioinformatics, 23* 127-128. doi:10.1093/bioinformatics/btl529
Letunic, I., & Bork, P. (2018). 20 years of the SMART protein domain annotation resource. *Nucleic Acids Res, 46* D493-D496. doi:10.1093/nar/gkx922
Luo, Y. J., Takeuchi, T., Koyanagi, R., Yamada, L., Kanda, M., Khalturina, M., . . . Satoh, N. (2015). The Lingula genome provides insights into brachiopod evolution and the origin of phosphate biomineralization. *Nat Commun, 6* 8301. doi:10.1038/ncomms9301
Marchler-Bauer, A., Bo, Y., Han, L., He, J., Lanczycki, C. J., Lu, S., . . . Bryant, S. H. (2017). CDD/SPARCLE: functional classification of proteins via subfamily domain architectures. *Nucleic Acids Res, 45* D200-D203. doi:10.1093/nar/gkw1129
Marchler-Bauer, A., Lu, S., Anderson, J. B., Chitsaz, F., Derbyshire, M. K., DeWeese-Scott, C., . . . Bryant, S. H. (2011). CDD: a Conserved Domain Database for the functional annotation of proteins. *Nucleic Acids Res, 39* D225-229. doi:gkq1189 [pii]
10.1093/nar/gkq1189
Mateo, L., & Gonzalez, J. (2014). Pogo-like transposases have been repeatedly domesticated into CENP-B-related proteins. *Genome Biol Evol, 6* 2008-2016. doi:10.1093/gbe/evu153
Moroz, L. L., Kocot, K. M., Citarella, M. R., Dosung, S., Norekian, T. P., Povolotskaya, I. S., . . . Kohn, A. B. (2014). The ctenophore genome and the evolutionary origins of neural systems. *Nature, 510* 109-114. doi:10.1038/nature13400
Murrell, B., Moola, S., Mabona, A., Weighill, T., Sheward, D., Kosakovsky Pond, S. L., & Scheffler, K. (2013). FUBAR: a fast, unconstrained bayesian approximation for inferring selection. *Mol Biol Evol, 30* 1196-1205. doi:10.1093/molbev/mst030
Nei, M., & Gojobori, T. (1986). Simple methods for estimating the numbers of synonymous and nonsynonymous nucleotide substitutions. *Mol Biol Evol, 3* 418-426. doi:10.1093/oxfordjournals.molbev.a040410
Punta, M., Coggill, P. C., Eberhardt, R. Y., Mistry, J., Tate, J., Boursnell, C., . . . Finn, R. D. (2011). The Pfam protein families database. *Nucleic Acids Res*. doi:gkr1065 [pii]
10.1093/nar/gkr1065
Punta, M., Coggill, P. C., Eberhardt, R. Y., Mistry, J., Tate, J., Boursnell, C., . . . Finn, R. D. (2012). The Pfam protein families database. *Nucleic Acids Res, 40* D290-301. doi:10.1093/nar/gkr1065
Putnam, N. H., Srivastava, M., Hellsten, U., Dirks, B., Chapman, J., Salamov, A., . . . Rokhsar, D. S. (2007). Sea anemone genome reveals ancestral eumetazoan gene repertoire and genomic organization. *Science, 317* 86-94. doi:10.1126/science.1139158

Quesneville, H., Nouaud, D., & Anxolabehere, D. (2005). Recurrent recruitment of the THAP DNA-binding domain and molecular domestication of the P-transposable element. *Mol Biol Evol, 22* 741-746. doi:10.1093/molbev/msi064

Roussigne, M., Kossida, S., Lavigne, A. C., Clouaire, T., Ecochard, V., Glories, A., . . . Girard, J. P. (2003). The THAP domain: a novel protein motif with similarity to the DNA-binding domain of P element transposase. *Trends Biochem Sci, 28* 66-69. doi:10.1016/S0968-0004(02)00013-0

Ryan, J. F., Pang, K., Schnitzler, C. E., Nguyen, A. D., Moreland, R. T., Simmons, D. K., . . . Baxevanis, A. D. (2013). The genome of the ctenophore Mnemiopsis leidyi and its implications for cell type evolution. *Science, 342* 1242592. doi:10.1126/science.1242592

Shi, M., Lin, X. D., Chen, X., Tian, J. H., Chen, L. J., Li, K., . . . Zhang, Y. Z. (2018). The evolutionary history of vertebrate RNA viruses. *Nature, 556* 197-202. doi:10.1038/s41586-018-0012-7

Siegmund, T., & Lehmann, M. (2002). The Drosophila Pipsqueak protein defines a new family of helix-turn-helix DNA-binding proteins. *Dev Genes Evol, 212* 152-157. doi:10.1007/s00427-002-0219-2

Simakov, O., Marletaz, F., Cho, S. J., Edsinger-Gonzales, E., Havlak, P., Hellsten, U., . . . Rokhsar, D. S. (2013). Insights into bilaterian evolution from three spiralian genomes. *Nature, 493* 526-531. doi:10.1038/nature11696

Srivastava, M., Begovic, E., Chapman, J., Putnam, N. H., Hellsten, U., Kawashima, T., . . . Rokhsar, D. S. (2008). The Trichoplax genome and the nature of placozoans. *Nature, 454* 955-960. doi:10.1038/nature07191

Srivastava, M., Simakov, O., Chapman, J., Fahey, B., Gauthier, M. E., Mitros, T., . . . Rokhsar, D. S. (2010). The Amphimedon queenslandica genome and the evolution of animal complexity. *Nature, 466* 720-726. doi:10.1038/nature09201

Suyama, M., Torrents, D., & Bork, P. (2006). PAL2NAL: robust conversion of protein sequence alignments into the corresponding codon alignments. *Nucleic Acids Res, 34* W609-612. Retrieved from http://www.ncbi.nlm.nih.gov/entrez/query.fcgi?cmd=Retrieve&db=PubMed&dopt=Citation&list_uids=16845082

Whelan, N. V., Kocot, K. M., Moroz, L. L., & Halanych, K. M. (2015). Error, signal, and the placement of Ctenophora sister to all other animals. *Proc Natl Acad Sci U S A, 112* 5773-5778. doi:10.1073/pnas.1503453112

Whelan, N. V., Kocot, K. M., Moroz, T. P., Mukherjee, K., Williams, P., Paulay, G., . . . Halanych, K. M. (2017). Ctenophore relationships and their placement as the sister group to all other animals. *Nat Ecol Evol, 1* 1737-1746. doi:10.1038/s41559-017-0331-3

Zhang, G., Fang, X., Guo, X., Li, L., Luo, R., Xu, F., . . . Wang, J. (2012). The oyster genome reveals stress adaptation and complexity of shell formation. *Nature, 490* 49-54. doi:10.1038/nature11413
