# Supplementary material for: Transposon-derived transcription factors across metazoans
Source: Front Cell Dev Biol. 2023 Mar 7;11:1113046. doi: 10.3389/fcell.2023.1113046 (PMC10027918; doi:10.3389/fcell.2023.1113046)
Supplement: Supplementary file 4 [file DataSheet1.PDF]

Supplementary Figures:

Transposon-derived transcription factors in Metazoa

Krishanu Mukherjee[1*], Leonid L. Moroz[1,2*]

[1]Whitney Laboratory for Marine Biosciences, University of Florida, St. Augustine, FL, 32080, USA; [2]Departments of Neuroscience and McKnight Brain Institute, University of Florida, Gainesville, FL, 32610, USA.

*****Corresponding authors

Emails:moroz@whitney.ufl.edu

https://orcid.org/0000-0002-1333-3176

krishanu@ufl.edu

Fig 1S. **Independent expansion and evolution of FLYWCH genes in ctenophores.**
This is a high-resolution picture of Fig. 2A (main text)

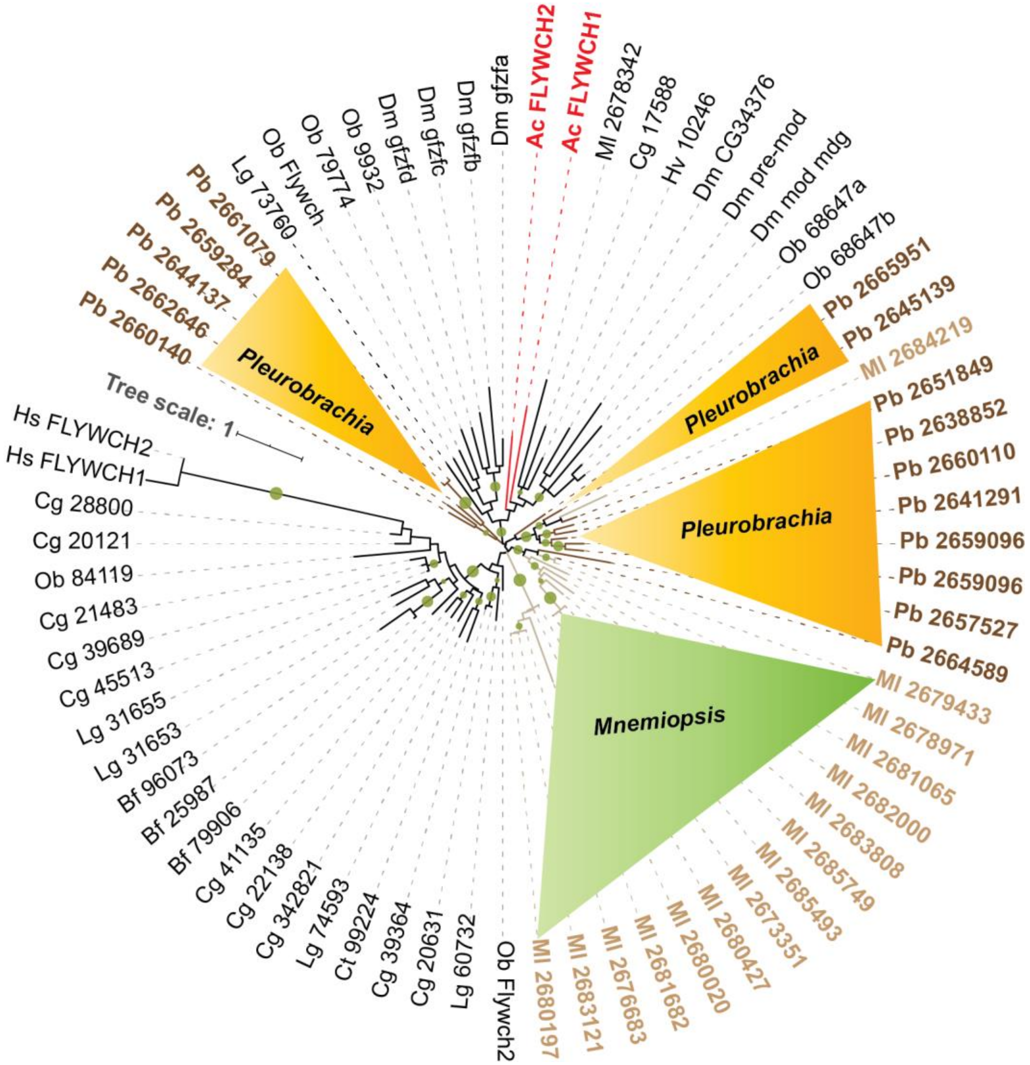

Maximum likelihood (ML) tree reconstructed using the FLYWCH domain and showing significant lineage-specific expansion in ctenophores. Both *Pleurobrachia* (yellow triangle) and *Mnemiopsis* (green triangle) showing the independent expansion of FLYWCH genes. Shimodaria-Hasegawa(Anisimova & Gascuel, 2006) (SH) supports of 80 or more are shown with green circles. The smallest purple circles indicate SH support of 80, while bigger circles are closer to 100. Excel File 1S contains gene IDs and sequences. Species abbreviations used in the tree are as follows: Ac - *Aplysia californica*, Ob - *Octopus bimaculoides*, Lg - *Lottia gigantea*, Cg - *Crassostrea gigas*, Ct - *Capitella teleta*, Dm - *Drosophila melanogaster*, Hv - *Hydra vulgaris*, Ml - *Mnemiopsis leydi*, Pb - *Pleurobrachia bachei*, Hs - *Homo sapiens*, Bf - *Branchiostoma floridae*.

Fig 2S. Independent expansion of THAP transcription factors across metazoans.

This is a high-resolution picture of Fig. 2B (main text)

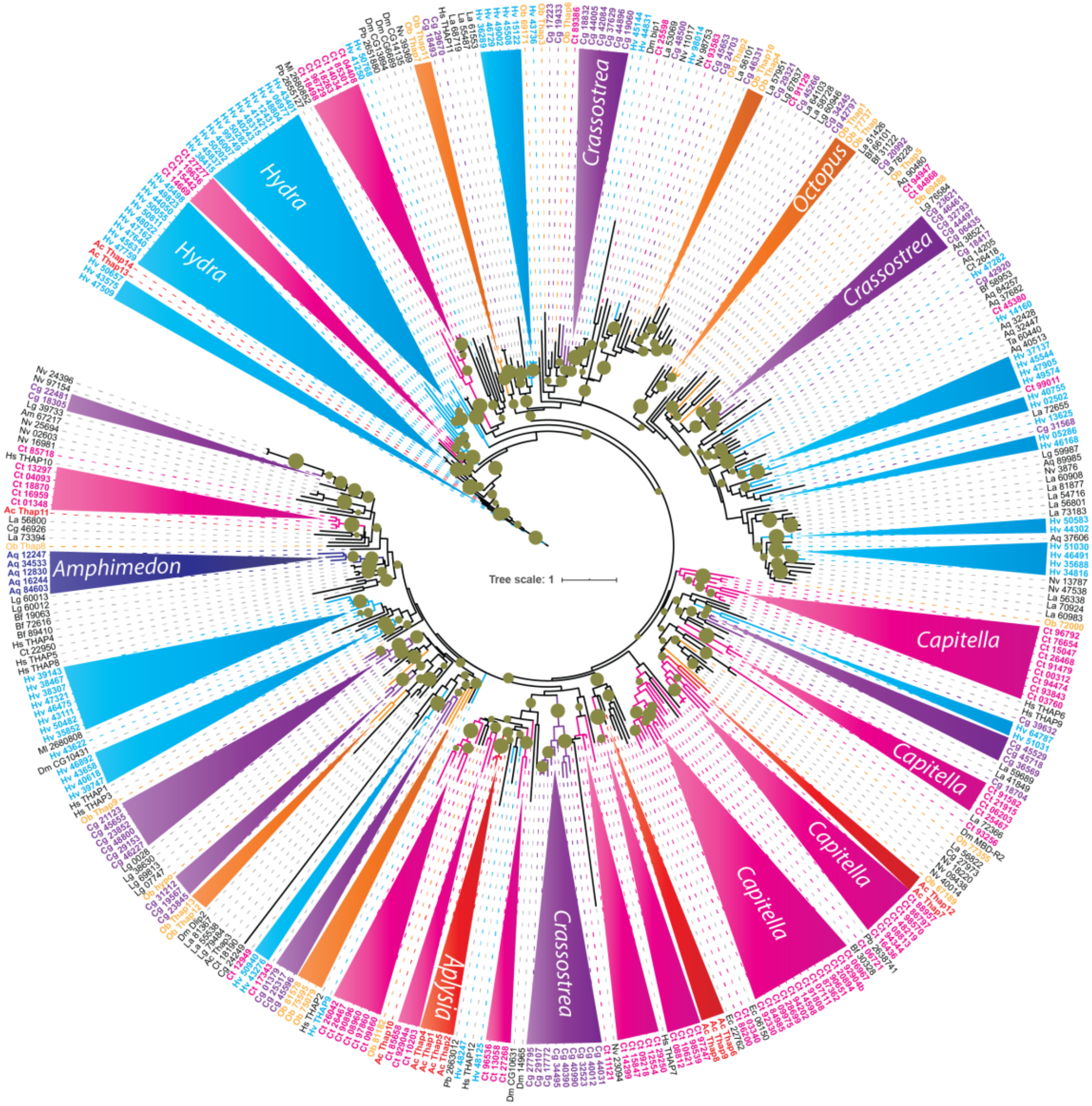

Maximum likelihood (ML) tree reconstructed using the THAP domains and illustrating lineage-specific expansion across metazoans. THAP-containing genes represent the most abundant and diverse superfamily of TE-derived TFs in Metazoa. For example, a remarkable diversification of THAP genes occurred in *Hydra* (Azure blue triangles), *Capitella* (pink triangles), *Crassostrea* (purple triangles), and *Aplysia* (red triangles). Orange triangles and fonts indicate the domestication events in *Octopus*, dark blue marks these events in the demosponge, *Amphimedon*. Shimodaria-Hasegawa(Anisimova & Gascuel, 2006) (SH) supports of 80 or more are marked. The smallest purple circles indicate SH support of 80, whereas bigger circles are closer to 100. Excel File 1S contains full species names, gene IDs, and sequences. See also Fig. 1S for abbreviations.

Fig 3S. Independent expansion and evolution HTH-Psq genes across metazoans.
This is a high-resolution picture of Fig. 2C

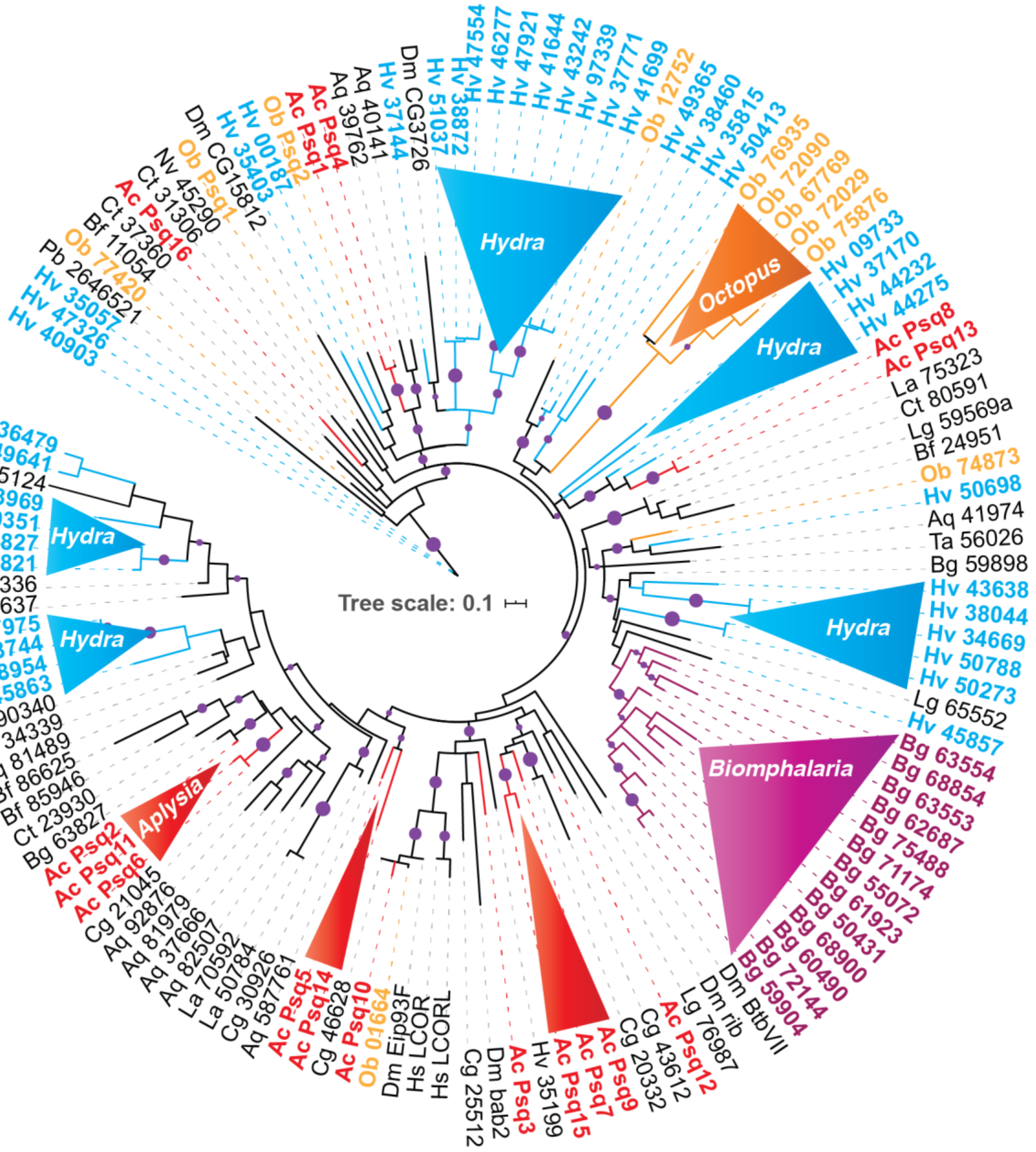

Maximum likelihood (ML) tree that shows the lineage-specific expansion of Psq domain TFs in Metazoa. The most prominent recruitments of HTH-Psq genes occurred in *Hydra* (Azure blue triangles), *Biomphalaria* (magenta triangles), *Octopus* (orange triangles), and *Aplysia* (red triangles). The tree also shows that none of the *Aplysia* genes have homologs in a related gastropod *Biomphalaria*. Shimodaria-Hasegawa(Anisimova & Gascuel, 2006) (SH) supports of 80 or more are marked. The smallest purple circles indicate SH support of 80, whereas bigger circles are closer to 100. Excel File 1S contains full species names, gene IDs, and sequences. See also Fig. 1S for abbreviations.

Fig 4S: Independent expansion of CENPB genes in *Amphimedon*, *Aplysia*, and *Drosophila*. This is the high-resolution picture of Fig. 2D

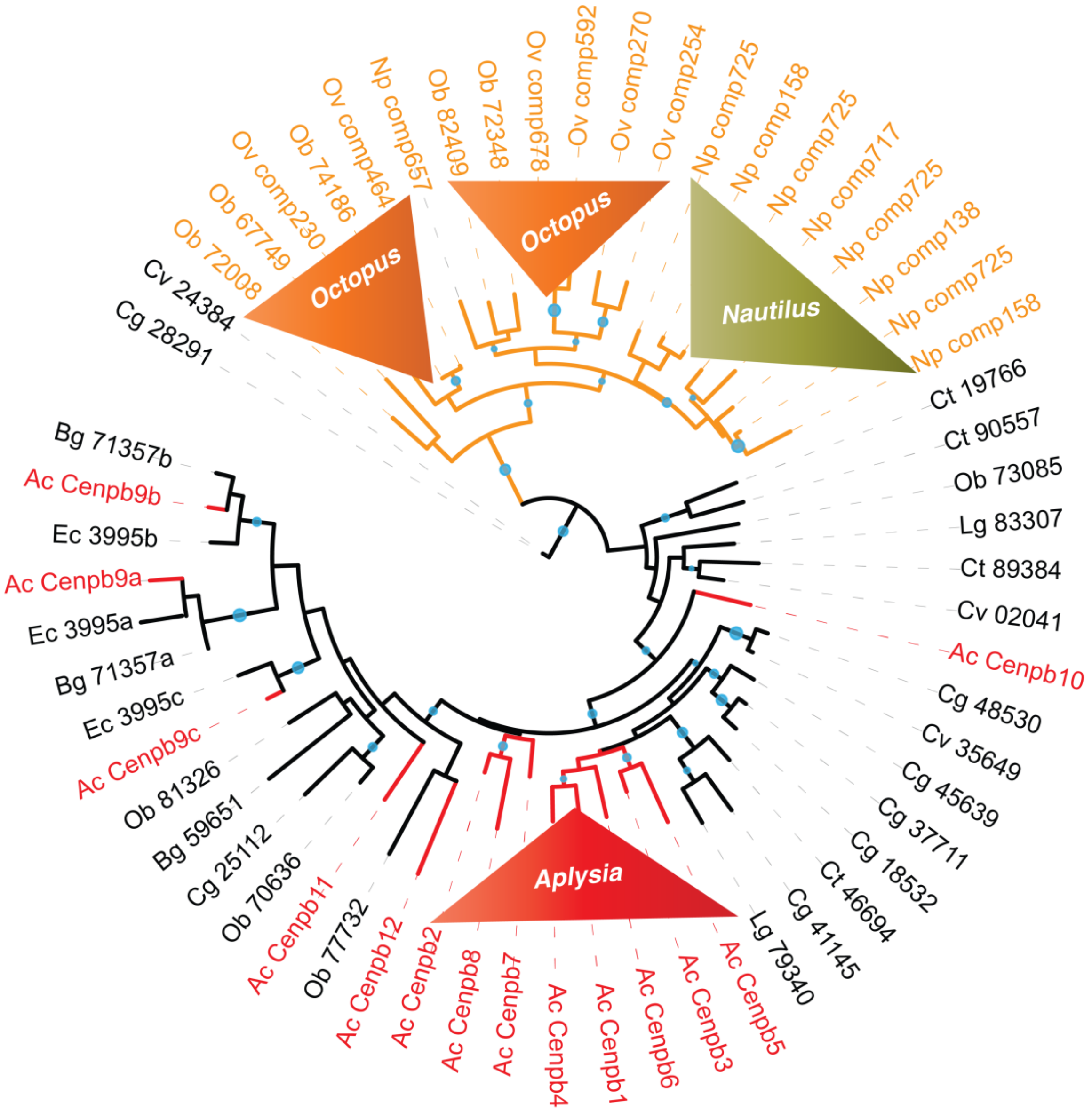

The maximum likelihood (ML) tree shows the lineage-specific expansion of the CENPB TFs in Metazoa. The reconstructed tree illustrates independent lineage-specific expansions of CENPB domain-containing TFs in *Octopus*, Nautilus, and *Aplysia*. *Aplysia* CENPB TFs are labeled with red color. *Nautilus* sequences were identified from the transcriptome sequenced in Moroz lab. Shimodaria-Hasegawa(Anisimova & Gascuel, 2006) (SH) supports of 80 or more are marked. The smallest purple circles indicate SH support of 80, whereas bigger circles are closer to 100. Excel File 1S contains full species names, gene IDs, and sequences. See also Fig. 1S for abbreviations.

Fig 5S: hAT transposon derived ZBED domestication across metazoans

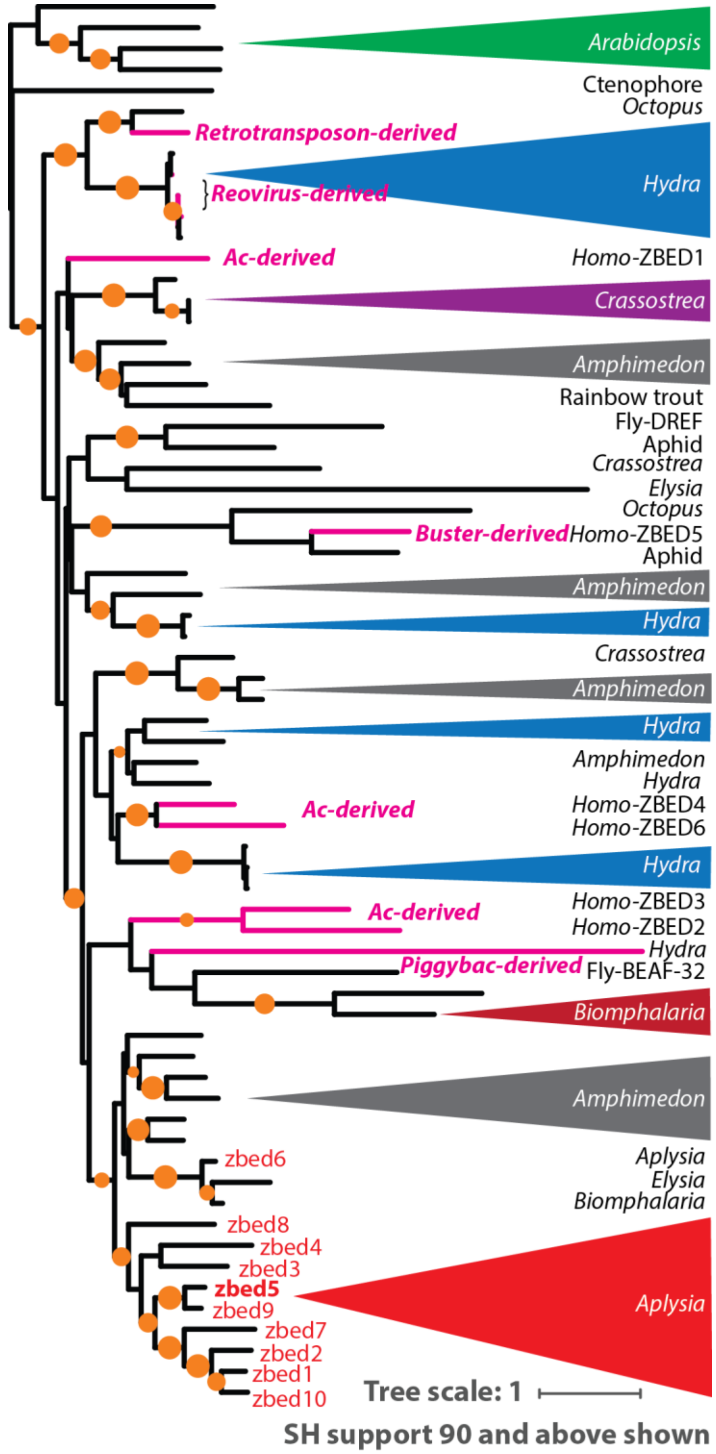

Maximum likelihood (ML) tree reconstructed using the ZBED domain illustrating independent domestication of ZBED TFs in *Aplysia* (2 events), *Biomphalaria* (2 events), *Crassostrea* (3 events), *Amphimedon* (5 events), and *Hydra* (6 events). Different

transposons gave rise to the distinct ZBED genes indicated by pink color and labeled next to the branches. SH supports of 90 or more are marked. The smallest purple circles indicate Shimodaria-Hasegawa(Anisimova & Gascuel, 2006) (SH) support of 90, whereas bigger circles are closer to 100. Excel File 1S contains gene IDs and sequences.

Notes: Aplysia ZBed5 TF, which is involved in neuroplasticity (see the text), is labeled bold. Ac-derived—*Ac activator transposable element* was the first transposable element recognized in maize(Mc, 1950). Piggyback (PB) transposon element efficiently transposes between vector to the host chromosome by unique cut-and-paste mechanism and was originally isolated from the genome of the moth *Trichoplusia*(Cary et al., 1989). Buster-derived—A subfamily of cut-and-paste DNA transposon derived from hAT transposable elements and are highly active in human cells(Li et al., 2013). Human ZBED gene ZBED5 was earlier shown to be related to the buster transposons and phylogenetically separated from the rest of the human ZBED genes(Hayward, Ghazal, Andersson, Andersson, & Jern, 2013). Retrotransposon derived—Retrotransposon or retrovirus are a type of genetic element that copy and paste themselves into the different genomic locations by converting RNA back to DNA(Dombroski et al., 1994). Reovirus derived—Similarly, while domain organization analysis 3 *Hydra* ZBED genes showed significant similarities with reovirus inner capsid protein lambda-1(Lemay & Danis, 1994).

Fig 6S: Independent expansion and evolution ZBED genes across metazoans

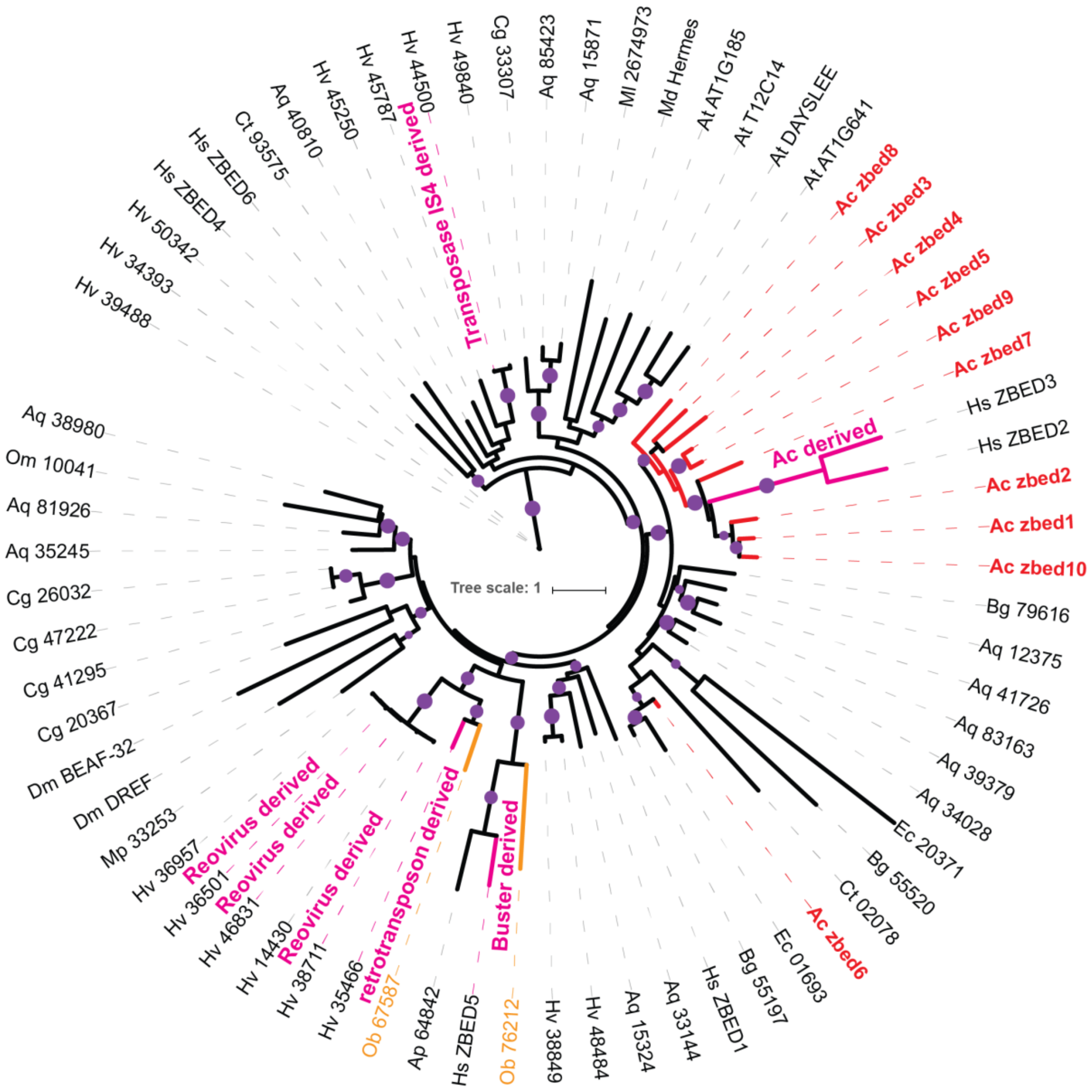

Maximum likelihood (ML) tree reconstructed from of the full-length ZBED-derived TFs. The tree indicates independent domestication in *Aplysia* and other metazoan species. Different transposon sources gave rise to the distinct ZBED genes marked by pink color and labeled next to the branches. *Aplysia* ZBED genes are shown in bold font and labeled with red color. Shimodaria-Hasegawa(Anisimova & Gascuel, 2006) (SH) supports of 90 or more are marked. The smallest purple circles indicate SH support of 90, whereas bigger circles are closer to 100. Excel File 1S contains full species names, gene IDs, and sequences. See also Fig. 1S for abbreviations.

Fig 7S: Independent expansion and evolution of CENPB genes across metazoans

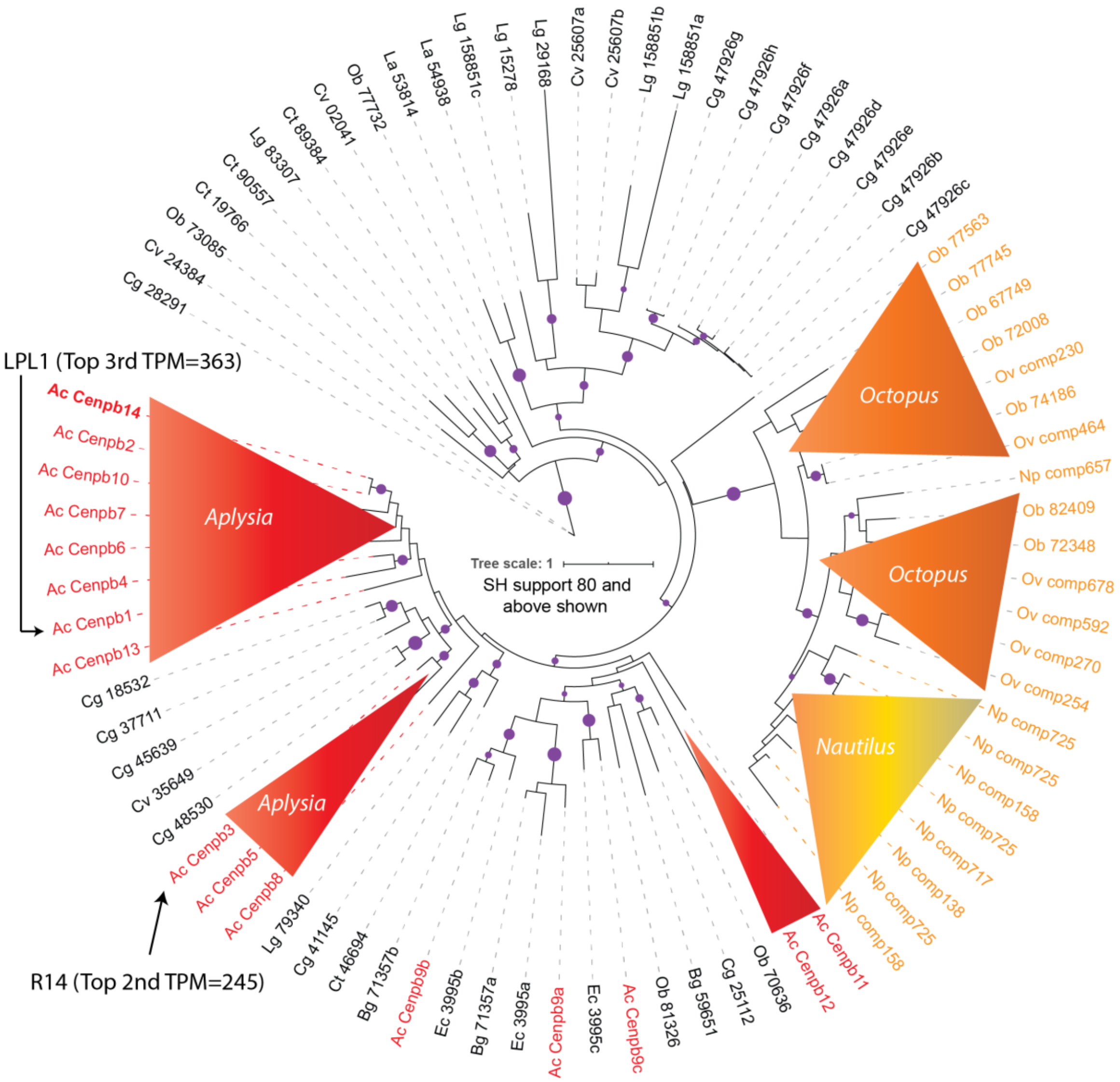

Phylogenetic analyses revealed convergent domestication of *Pogo* transposon derived CENPB TFs in *Aplysia* (red triangle), *Octopus* (orange triangle), and *Nautilus* (yellow triangle). Some of the CENPB TFs (arrows) in *Aplysia* show the strongest expression pattern in individual neurons. They have the top 2nd and top 3rd expression label of all the TFs identified in the *Aplysia californica* genome. Within the Cephalopod lineage, we identify two distinct events of *Pogo* domestication: one in the lineage leading to *Nautilus pompilius* (Np) (shown with yellow triangle) and another incident that occurred during *Octopus* evolution (shown with the orange triangle). File 1S contains full species names, gene IDs, and sequences. Abbreviations for species on this tree: Ac - *Aplysia californica*, - Lg – *Lottia gigantea*, Ct – *Capitella teleta*, Cg - *Crassostrea gigas*, Cv - *Crassostrea virginica*, Bg – *Biomphalaria glabrata*, Ov –*Octopus vulgaris*, Ec – *Elysia chlorotica*, La - *Lingula anatina*. Shimodaria-Hasegawa(Anisimova & Gascuel, 2006) (SH) support of 80 or more is shown. The smallest purple circles indicate SH support of 80, while bigger circles are closer to 100. *Aplysia* Cenpb14 – the TF associated with neuroplasticity is shown in bold.

Fig 8S: Independent expansion and evolution of FHY3 genes across metazoans

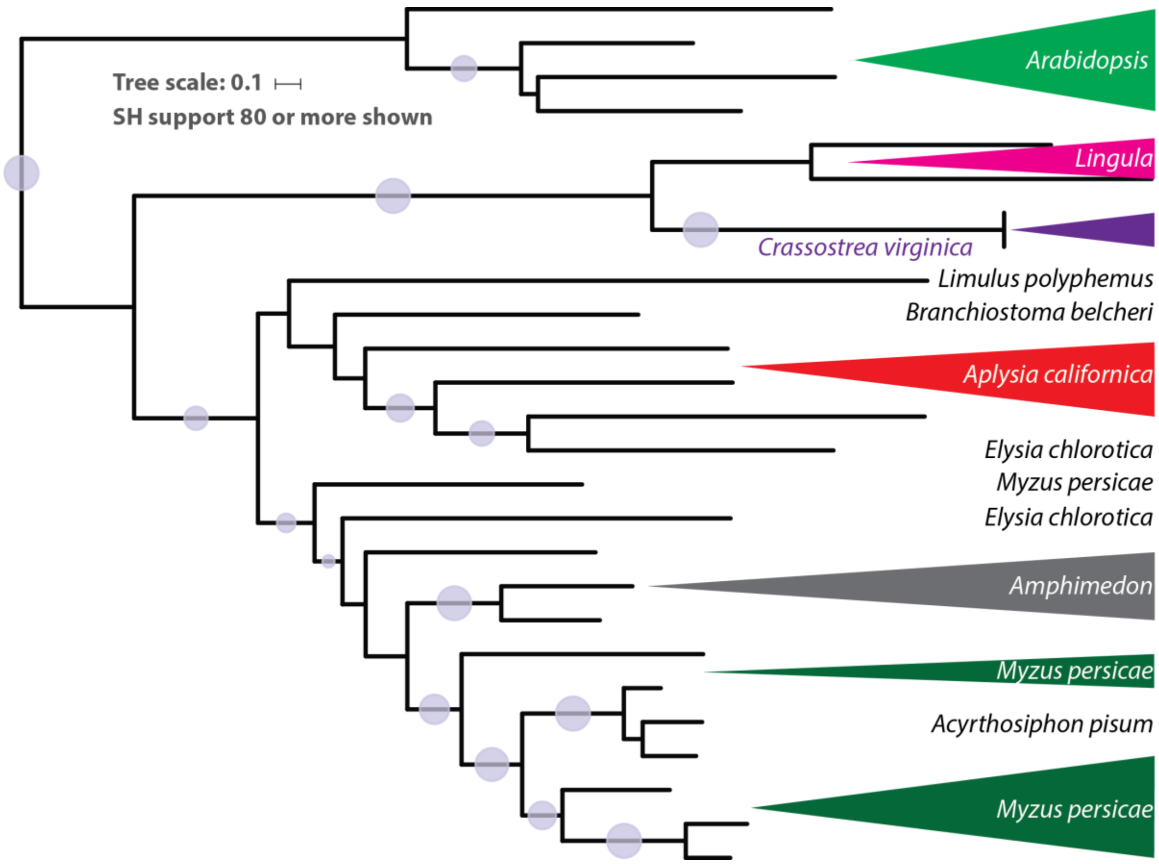

Phylogenetic analyses revealed convergent domestication of *MULE* transposon-derived FHY3 TFs in *Aplysia* and other species. SH supports of 80 or more are marked. The smallest purple circles indicate SH support of 80, whereas bigger circles are closer to 100. Excel File 1S contains gene IDs and sequences.

References to the supplementary figures

Anisimova, M., & Gascuel, O. (2006). Approximate likelihood-ratio test for branches: A fast, accurate, and powerful alternative. *Syst Biol, 55*(4), 539-552. doi:10.1080/10635150600755453

Cary, L. C., Goebel, M., Corsaro, B. G., Wang, H. G., Rosen, E., & Fraser, M. J. (1989). Transposon mutagenesis of baculoviruses: analysis of Trichoplusia ni transposon IFP2 insertions within the FP-locus of nuclear polyhedrosis viruses. *Virology, 172*(1), 156-169. doi:10.1016/0042-6822(89)90117-7

Dombroski, B. A., Feng, Q., Mathias, S. L., Sassaman, D. M., Scott, A. F., Kazazian, H. H., Jr., & Boeke, J. D. (1994). An in vivo assay for the reverse transcriptase of

human retrotransposon L1 in Saccharomyces cerevisiae. *Mol Cell Biol, 14*(7), 4485-4492. doi:10.1128/mcb.14.7.4485

Hayward, A., Ghazal, A., Andersson, G., Andersson, L., & Jern, P. (2013). ZBED evolution: repeated utilization of DNA transposons as regulators of diverse host functions. *PLoS One, 8*(3), e59940. doi:10.1371/journal.pone.0059940

Lemay, G., & Danis, C. (1994). Reovirus lambda 1 protein: affinity for double-stranded nucleic acids by a small amino-terminal region of the protein independent from the zinc finger motif. *J Gen Virol, 75 (Pt 11)*, 3261-3266. doi:10.1099/0022-1317-75-11-3261

Li, X., Ewis, H., Hice, R. H., Malani, N., Parker, N., Zhou, L., . . . Craig, N. L. (2013). A resurrected mammalian hAT transposable element and a closely related insect element are highly active in human cell culture. *Proc Natl Acad Sci U S A, 110*(6), E478-487. doi:10.1073/pnas.1121543109

Mc, C. B. (1950). The origin and behavior of mutable loci in maize. *Proc Natl Acad Sci U S A, 36*(6), 344-355. doi:10.1073/pnas.36.6.344
